# Supplementary material for: Comparing kinematic and kinetic demands on the knee joint during selected physiotherapy exercises and activities of daily living
Source: Technol Health Care. 2026 Mar 31;34(3):408–21. doi: 10.1177/09287329251413413 (PMC13195061; doi:10.1177/09287329251413413)
Supplement: sj-zip-1-thc-10.1177_09287329251413413 - Supplemental material for Comparing kinematic and kinetic demands on the knee joint during selected physiotherapy exercises and activities of daily living [file sj-zip-1-thc-10.1177_09287329251413413.zip › Manuscript_Review_Highlighted.docx]

Comparing kinematic and kinetic demands on the knee joint during selected physiotherapy exercises and activities of daily living

# Abstract

**Background**

The knee is one of the most common areas to suffer injuries or be affected by surgery. Physiotherapy rehabilitation was shown to support recovery, but evidence guiding optimal rehabilitation practices is limited. To recommend appropriate exercises, it is essential to understand the musculoskeletal requirements involved in both physiotherapy and activities of daily living (ADLs).

**Objective**

This study aimed to evaluate and compare the knee joint kinematics, joint forces and muscle activity in knee flexors and extensors during selected rehabilitation exercises and ADLs.

**Methods**

Kinematic and kinetic data from 30 healthy participants were collected during 20 different tasks. Full-body musculoskeletal simulations were performed to estimate peak knee joint angles, angular velocities, joint reaction forces, and muscle activity of the knee flexors and extensors.

**Results**

Comparatively high requirements were observed for lunges, squats, stair walking and gait. Medium requirements were observed for sitting down and rising from a chair. Low requirements were observed for balance shifts and variations of the single leg stand.

**Conclusion**

Overall, ADLs like gait and stair walking show surprisingly high requirements compared to many exercises employed in physiotherapy. These findings are a step towards biomechanically informed exercise selection and the development of personalized rehabilitation programs.

# Keywords

Personalized Rehabilitation, Musculoskeletal Simulation, Knee Kinematics, Knee Kinetics, Muscle Activity

# Introduction

The knee joint is one of the most common areas to suffer injuries. Reported prevalence of knee joint injuries ranges between 10% and 25% [1]. Additionally osteoarthritis of the knee joint is a common, joint disease [2, 3]. Projections based on current demographic and epidemiological trends indicate a rising prevalence of knee osteoarthritis in the future [4]. Accordingly, the prevalence of total knee arthroplasty (TKA) as one of the most effective interventions is also expected to rise [5–8].

This indicates an increased demand for rehabilitation programs focused on regaining knee joint functionality after injury or surgical intervention. By utilising physiotherapy, short term improvements regarding surgery outcomes such as pain, physical function and range of motion can be achieved [9–11]. However, while different rehabilitation modalities result in comparable improvements, there is a lack of evidence regarding the most favourable rehabilitation practise following injury or surgical intervention [12–14].

An ideal rehabilitation program should follow a progressive approach by gradually increasing the intensity of exercises, guiding the patient through increasingly demanding tasks [15]. To ensure effectiveness and safety, the program must be tailored to the patient’s current physical capabilities, allowing for appropriate training stimulus without exceeding their limits [15]. A major challenge in rehabilitation is delivering an effective training stimulus without risking overload or setbacks.

To develop a rehabilitation program with progressively increasing intensity, it is essential to understand the demands associated with different exercises. In addition, knowledge of the demands experienced during activities of daily living (ADLs) is crucial. ADLs contribute to the cumulative joint loading throughout the day and represent a key target in rehabilitation, as restoring the ability to perform these tasks is a primary goal for many patients [16, 17]. An ideal rehabilitation program progressively guides patients through increasingly demanding tasks, with the goal of enabling safe and effective performance of ADLs.

Thus, assessing and comparing the demands associated with commonly used physiotherapy exercises and common ADLs can provide a foundation for developing such progressive programs. Ranking these tasks according to selected parameters can offer therapists additional guidance in selecting the appropriate task, based on a patient’s performance during other tasks. The demand of a task – i.e. the requirements that need to be met by the patient to safely engage in the task - and the outcome targeted by the task are defined by several parameters:

Joint forces are integral to define the experienced loading. The magnitude and direction of the force between the femoral condyles and tibial plateau determine which type of loading is experienced, like compressive force, distractive force or shear force. This in turn determines which anatomical structures such as cartilage and ligaments are loaded.

Additionally, altered kinematics - specifically reduced peak knee joint angles – were shown in TKA patients up 18 months post-surgery [18–20]. Similarly, abnormal knee kinematics including knee flexion and flexion velocity were observed up to one year after anterior cruciate ligament reconstruction [21, 22]. At the same time however, increasing range of motion is an important expectation for many patients [16, 17]. Accordingly, the range of motion and angular velocity during a task have important implications for its use in rehabilitation.

Furthermore, surgery or injury often results in strength deficits, which can hinder functional recovery [23–26]. Strength training has been shown to improve functional outcomes by addressing these deficits and enhancing muscular performance [27]. Selecting exercises with appropriate muscular demands can help ensure targeted and effective strength recovery.

While measurements/predictions of joint kinematics, joint forces, and muscle activity are available for a wide range of tasks (e.g. [28–55]), only a limited number of studies have directly compared these parameters across rehabilitation tasks (e.g. [39, 41]). Comprehensive overviews comparing multiple parameters across both exercises and ADLs remain scarce, highlighting the need for studies that offer such a comparison to support biomechanically informed rehabilitation planning. This study aimed to evaluate and compare the peak knee joint angles and angular velocity, peak knee joint forces and peak muscle activity in knee flexors and extensors during selected rehabilitation exercises and ADLs.

# Methods

## Participants

Healthy adults (n = 30; 8 male, 22 female; age, height, and body mass reported as mean ± SD: 22.8 ± 2.1 years, 1.72 ± 0.08 m, 67.9 ± 10.5 kg) with no musculoskeletal injuries, neurological disorders or degenerative joint diseases, were recruited for this study. All participants provided written informed consent, and the study was approved by the local university's ethics committee (approval no: 23-3260-101).

## Data acquisition

Motion data and external forces acting on the subject from the environment were collected for 20 different tasks (Table 1). Before each task, subjects watched an instructional video explaining how to perform the task. The video could be watched several times if required. No further instructions were given. Since no actual patients were assessed, the right leg was designated as the leg of interest.

The recording sessions were split into two blocks: Block one included tasks using motion capture alone and in combination with a staircase, while block two focused on tasks performed with a chair. Within each block, the order of the tasks was randomly selected.

The recording sessions were split into two blocks each using motion capture

Table 1: Description of the performed tasks. Each recording session was split into two blocks: (1) First block; (2) Second block. Within each block, the order of the tasks was randomly selected for each subject. *Ground reaction forces were not recorded but derived from full-body motion using a so-called ground reaction force prediction method [56].

| **Task** | **Description** |
| --- | --- |
| Gait (Gait) ^(1), *^ | Subjects were walking at a self-selected pace along a seven meters long track. |
| Long arc quad (LAQ) ^(2), *^ | Subjects started in a sitting position and extended their leg at the knee. After reaching maximum knee extension, the leg was brought back down. |
| Lunge (Lunge) ^(1), *^ | Subjects performed a forward lunge with the right leg in front. |
| Squat (Squat) ^(1), *^ | Subjects performed a free squat with their hands at their hips and the feet at shoulder width. |
| Single leg stand (SLS) ^(1), *^ | Subjects balanced on their right leg for 30 seconds. |
| Advanced single leg stand (SLSA) ^(1), *^ | Subjects balanced on their right leg for 30 seconds with their eyes closed. |
| Supported single leg stand (SLSS) ^(1), *^ | Subjects balanced on their right leg for 30 seconds, while resting their hand on the handrail of a staircase for support. |
| Sitting down on a chair (SitDown) ^(2)^ | Subjects stood in front of a chair facing away with their knees fully extended, and then sat down without using the armrests. |
| Standing up from a chair (StandUp) ^(2)^ | Subjects rose from a chair to a standing position without using the armrests. |
| Supported sitting down on a chair (SitDown_S) ^(2)^ | Subjects stood in front of a chair facing away with their knees fully extended, and then sat down using the armrests. |
| Supported standing up from a chair (StandUp_S) ^(2)^ | Subjects rose from a chair to a standing position using the armrests. |
| Climbing up one step (StepUp) ^(1)^ | Subjects stood on the lower instrumented step of the staircase facing upwards. They then brought their right leg up to the next higher step before following with the left leg. |
| Reversing down one step (StepUpReverse) ^(1)^ | Subjects stood on the upper instrumented step of the staircase facing upwards. They then brought their left leg down to the next lower step before following with the right leg. |
| Step down exercise (StepDown) ^(1)^ | Subjects stood on the upper instrumented step of the staircase facing downwards. They then brought their left leg down to slightly touch the next lower step before returning to the starting position. |
| Stair walking downwards (StairDown) ^(1)^ | Subjects walked down the staircase without using the handrail. |
| Stair walking upwards (StairUp) ^(1)^ | Subjects walked up the staircase without using the handrail. |
| Supported stair walking downwards (StairDown_S) ^(1)^ | Subjects walked down the staircase while using the handrail. |
| Supported stair walking upwards (StairUp_S) ^(1)^ | Subjects walked up the staircase while using the handrail. |
| Frontal balance shift (Bal.Front.) ^(1), *^ | Subjects started in a neutral stand with their feet at shoulder width and their hands at their hips. They then shifted their weight from the left to the right leg five times. |
| Sagittal balance shift (Bal.Sag.) ^(1), *^ | Subjects stood in a stride position with the right foot in front of the left and their hands at their hips. They then shifted their weight from the front to the rear leg five times. |

## Experimental set up

Motion data was recorded using a marker-less motion capture system (CapturyLive v 255, TheCaptury, Germany). Data was simultaneously recorded with eight machine vision cameras (FLIR Blackfly S16S2C, Teledyne FLIR LLC, USA) at 60 Hz. This configuration demonstrated good agreement compared to a marker-based gold standard system with a mean absolute error of 3.5° for knee flexion [57].

A six-step staircase with two instrumented steps was built (Figure 1). Six wooden steps were mounted on an aluminium frame. The dimensions were chosen to comply with German regulatory requirements for the design of stairs in public buildings [58]. Two force plates (9260AA6, Kistler Instrumente AG, Switzerland) were mounted on the third and fourth step. Ground reaction forces were recorded at 600 Hz. A handrail was connected to the main frame at a height of 125 cm via an assembly of three uni-axial load cells (PW10AC3MR, Hottinger Brüel & Kjaer GmbH, Germany) oriented perpendicular to each other (Figure 1). The force applied to the handrail was recorded at 300 Hz.

*[Insert figure 1; Caption: “Figure 1: Experimental set up used to record interface forces between the human body and the environment during physiotherapy exercises and activities of daily living. (A) Staircase with force plates on the third and fourth step and load cells connecting the right handrail to the frame; (B) Chair with force plates in front and load cells connecting the armrests to the frame.”]*

Additionally, an instrumented chair was built (Figure 1). A wooden baseplate was mounted on an aluminium frame. The arm rests were connected to the frame via the previously mentioned assembly of three uni-axial load cells. The force applied to the arm rests was recorded at 300 Hz. Two force plates (9260AA6, Kistler Instrumente AG, Switzerland) were positioned in front of the chair to capture ground reaction forces at a rate of 600 Hz. The chair height was 45 cm, a typical height for biomechanical studies on sit-to-stand movements [59].

## Musculoskeletal modelling

Recorded kinematic and kinetic data were used as input for musculoskeletal simulations using The AnyBody Modeling System (v 7.4.4, AnyBody Technology A/S, Denmark). A custom model based on the full-body ‘Plug-in-gait_Simple’ model from the AnyBody Managed Model Repository (v 2.4.4, AnyBody Technology, Denmark) that was adapted to the input from the selected motion capture system was used [57]. This model features a lower extremity model with 6 degrees of freedom and 55 muscles divided into 169 elements per leg [60]. A 3-element muscle model which includes the force-length and force-velocity relationship was selected. Maximum isometric strength of the muscles was scaled according to subject height and weight based on a regression law [61]. The muscle redundancy was solved using a polynomial muscle recruitment algorithm with power 𝑝 = 2.

The default low-pass filters settings with a cut-off frequency of 5 Hz and 12 Hz for kinematic and kinetic input respectively were applied. The model was scaled to each subjects’ dimensions and subsequently the MarkerTracking and InverseDynamics operation were run. In total 600 simulations were batch processed using Python (v 3.9.12, Python Software Foundation, USA) and AnyPyTools (v1.11.0) [62].

## Data analysis

Output from simulations was processed using custom Python (v 3.9.12) scripts. Knee joint flexion and flexion velocity, joint forces and muscle activity were analysed. Normality was assessed using the Shapiro–Wilk test with a significance level of 5%. Since many of the results are not normally distributed, the median and interquartile ranges are reported. For each parameter, tasks were ranked in descending order based on the predicted peak values, and adjacent tasks were compared to identify significant differences. The Wilcoxon signed-rank test was used for pairwise comparisons. A 5% significance level was applied, and Bonferroni correction was used to account for multiple comparisons.

For one subject the StepDown task and for four subjects the gait trials were excluded from the analysis due to numerical instabilities during simulation.

# Results

Figures 2, 3 and 4 show the knee angle, angular velocity, component wise joint force and muscle activity in knee flexors and extensors over the entire movement cycle for all tasks.

*[Insert figure 2; Caption: “Figure 2: Profiles of knee joint angle and joint angular velocity over the entire movement cycle for all tasks. Lines show the median over all subjects. The shaded areas show the interquartile range.”]*

*[Insert figure 3; Caption: “Figure 3: Profiles of anterior-posterior, medio-lateral and proximo-distal knee joint force over the entire movement cycle for all tasks. Lines show the median over all subjects. The shaded areas show the interquartile range.”]*

*[Insert figure 4; Caption: “Figure 4: Profiles of quadriceps and hamstring muscle activity over the entire movement cycle for all tasks. Lines show the median over all subjects. The shaded areas show the interquartile range.”]*

Joint kinematics (Figure 2) revealed clear angular excursions and velocity peaks in dynamic tasks (e.g., Gait, stair walking, squatting), while static balance tasks exhibited minimal angular change.

Joint force profiles (Figure 3) illustrate both tasks with relatively constant loading (e.g., single leg stand) and tasks characterized by more pronounced fluctuations. In some cases, peak forces occur only briefly (e.g., stair walking, Gait), whereas in others they are sustained over a longer duration (e.g., StepDown, Lunge, Squat).

Muscle activity (Figure 4) shows distinct task-dependent patterns, with quadriceps activation peaking during gait and stair walking, while reaching longer sustained peak activity during StepDown, Lunge and Squat. Hamstring activation remained comparatively lower.

Figure 5 shows the median of the maximum knee flexion and extension angle, as well as flexion and extension angular velocity for all tasks.

*[Insert figure 5; Caption: “Figure 5: Maximum knee flexion(A), knee extension (B), knee flexion velocity (C) and knee extension velocity (D) for all tasks. Median and interquartile range are presented. Significant differences between adjacent tasks are shown using Bonferroni-corrected p-values (n = 19 comparisons).”]*

Knee flexion was highest for lunges (116° [112°, 123°]) and squats (115° [99°, 128°]), while balance shifts and single leg stand variations showed values below 20°. Maximum knee extension ranged from -4° [-8°, -1°] during Bal.Sag. shifts to -15° [-20°, -11°] for StairDown.

Flexion/extension velocity was highest during gait and stair exercises, with maximum flexion velocity at 338°/s [301°/s, 373°/s] during StairUp and maximum extension velocity at 331°/s [316°/s, 364°/s] for gait. The lowest velocities were seen during balance shifts and single leg stands, with flexion reaching 20°/s and extension reaching 18°/s.

Figure 6 shows the median of the maximum knee joint forces, normalized to body weight (BW) acting for all tasks.

*[Insert figure 6; Caption: “Figure 6: Maximum posterior component (A), anterior component (B), medial component (C), lateral component (D), distal component (E) and proximal component of the force acting on the knee joint for all tasks. Forces are normalized to body weight (BW) and expressed in a coordinate system aligned with the tibial plateau (see sub-figure A). Median and interquartile range are presented. Significant differences between adjacent tasks are shown using Bonferroni-corrected p-values (n = 19 comparisons).”]*

The highest posterior shear force occurred during gait (0.4 BW [0.4 BW, 0.6 BW]) while the lowest force was seen for supported (-0.1 BW [-0.2 BW, -0.1 BW]) and unsupported (-0.1 BW [-0.2 BW, -0.1 BW]) stair ascent with values below zero indicating an anterior force.

Anterior shear forces ranged from 0.9 BW [0.8 BW, 1.0 BW] for lunges to 0.1 BW [0.1 BW, 0.1 BW] for Bal.Front.

During gait, a medial shear force of 0.4 BW [0.3 BW, 0.5 BW] was observed. For all other tasks, medial shear ranged between 0.1 BW and -0.1 BW.

Lateral shear was highest for lunges (1.3 BW [1.2 BW, 1.5 BW]) and lowest for balance shifts, single leg stands, and LAQ (≤ 0.2 BW).

Axial forces in the distal direction reached the highest values of all force directions with up to 6.6 BW [6.0 BW, 7.2 BW] for lunges. Except for LAQ with 0.9 BW [0.7 BW, 1.0 BW], all tasks reached forces above 2 BW.

In the proximal direction, only negative forces were observed, indicating that in the axial direction only distal forces occur. The highest proximal force i.e. the lowest minimum distal force was observed for Bal.Front. (-0.3 BW [-0.3 BW, -0.2 BW]). The lowest proximal force i.e. the highest minimum distal force was observed for StepDown (-1.9 BW, [-2.1 BW, -1.4 BW]).

Figure 7 shows the median of the maximum muscle activity of the quadriceps and the hamstring muscle for all tasks.

*[Insert figure 7; Caption: “Figure 7: Maximum muscle activity in the quadriceps (A) and hamstring (B) muscle for all tasks. Median and interquartile range are presented. Significant differences between adjacent tasks are shown using Bonferroni-corrected p-values (n = 19 comparisons).”]*

Quadriceps activity was highest at 68% [60%, 81%] for lunges. The lowest values, all below 10%, were seen for balance shifts, single leg stands, and LAQ. Hamstring activity was highest during gait (42% [32%, 47%]), while LAQ showed the lowest activity at 3% [2%, 5%].

Figures and videos visualizing all parameters per exercise can be found in the supplementary material.

# Discussion

Following knee injury/surgery, patients typically receive physiotherapy as part of their rehabilitation. Postoperative physiotherapy positively impacts patient outcomes [13, 14, 63]. Still, there is no conclusive evidence about which exercise program results in the most favourable outcomes [13, 14, 63]. This study aimed to assess the minimum requirements during different exercises and ADLs to help establish a foundation for biomechanically informed exercise selection.

Tasks like lunges, squats, gait, and stair walking generally showed high demands regarding joint kinematics, joint forces and muscle activity. In contrast, balance shifts and single leg stand variations exhibited comparatively lower requirements.

LAQ requires high ROM while the angular velocities are moderate, and the joint forces and muscle activity is low. This points to the potential of the exercise as a mobilization exercise with comparatively low joint loading.

Balance shifts have low requirements regarding ROM and angular velocity. Maximum joint force is also mostly low except for the distal force. As these forces are lower than those during gait and stair walking, balance shifts can be used to train the knee towards the axial loading experienced during these tasks while simultaneously requiring only low muscle activity.

The single leg stand variations also require little ROM and low angular velocities. Maximum joint force is again small except for the distal direction. This agrees with in vivo measurements of knee joint force of 2.55 BW during single leg stand that is dominated by the axial component [28]. Both quadriceps and hamstring activity are, while still moderate, higher compared to balance shifts. Similar levels of thigh muscle activation during single leg standing were found using electromyography (EMG) measurements [54, 55].

The different variations of SitDown/StandUp require high ROM and moderate flexion/extension velocities. Similar knee extension velocity during sit-to-stand was previously reported [64]. The distal force during the supported variation is comparable to single leg stands and balance shifts at 2.6 BW and 2.8 BW. During the unsupported variation, distal force reaches 3.7 BW and 3.8 BW respectively, still lower when compared to gait and stair walking related tasks. Shear forces in all directions range from low to moderate when compared with other tasks, making for an overall moderate joint loading profile. The joint forces in the current study are higher compared to previously reported in vivo measurements but similar to model estimations [28, 34, 39, 40, 49]. With moderate quadriceps activity and low hamstring activity, SitDown/StandUp can be used for strengthening knee extensors while putting very little demand on knee flexors. However, muscular demand might differ in patient cohorts due to reduced muscle strength. For example, in TKA patients, knee extensor activity of up to 39% and knee flexor activity of up to 19% was found [48].

Interestingly, gait, one of the main tasks of human movement, is one of the most demanding tasks, with respect to the parameters examined in this study. The knee flexion and extension velocity are high compared to many exercises. Furthermore, the highest posterior and medial shear was found during gait. The distal force during gait is almost as high as during some stair walking related tasks. Quadriceps activity during gait also comparable to stair walking, while hamstring activity was highest among all tasks. EMG measurements during gait range from 10% to 58% for knee extensors and 10% to 52.6% for knee flexors [35–38]. Factors like study population, walking speed, and foot strike pattern might influence results and complicate comparisons [35–38].

Increasing walking distance is a primary patient expectation [28,29]. Accordingly, rehabilitation should aim to restore gait - thus, placing high demands on rehabilitation programs to successfully engage in such highly demanding tasks post-rehabilitation.

During stair walking, the maximum knee flexion and extension are low compared to other tasks, yet the highest knee flexion velocity in this study was seen for the supported and unsupported upwards stair walking. During supported and unsupported downwards stair walking, the second highest and third highest knee extension velocity is reached. Even with sufficient ROM, the required angular velocities must be achieved. In this study, stair walking showed higher knee flexion (upstairs) and extension (downstairs) velocities than related exercises, suggesting these exercises may not train patients for the actual velocities needed. However, older adults or patients may navigate stairs slower, leading to lower angular velocities during stair walking compared to related exercises.

Distal joint forces are high during stair walking, with only lunges and the StepDown task demonstrating higher loading. During stair ascent high anterior shear is experienced while during stair descent high posterior shear is experienced indicating an overall high joint loading profile.

The quadriceps activity during stair walking, is comparable to gait, but still considerably lower when compared to lunges, squats and StepDown indicating that patients who can perform these exercises display sufficient quadriceps strength for stair walking. Regarding hamstring activity, a clear distinction between upwards and downwards stair walking can be seen. Overall, the muscle activity values found in this study fall within the range of reported EMG measurements during stair walking [44–47].

Considering all parameters, stair walking ranks as one of the more demanding tasks. Yet, similarly to gait, for many patients being able to successfully navigate stairs is one of their main expectations, again stressing the need for effective rehabilitation [16, 17].

The joint loading during gait and stair walking found in this study is higher than previous in vivo measurements but aligns more closely with model estimated joint forces: For gait a maximum axial knee joint force of 2.08 BW – 2.76 BW and maximum resultant force of 2.61 BW – 3.1 BW is reported [28, 29, 32, 34, 40]. Model estimated axial forces range from 2.5 BW to 5.79 BW while resultant forces range from 2.97 BW to 3.33 BW [30, 31, 33, 39, 42].

During stair ascent, in vivo measurements range from 2.5 BW to 3.06 BW for axial joint forces while up to 4 BW are reported for resultant forces [28, 29, 32, 40]. Model estimated axial forces reach 3.9 BW while resultant forces range from 3.6 BW to 5.88 BW [33, 39, 43]. During stair descent, in vivo measurements range from 3.27 BW to 3.52 BW for axial joint forces and from 3.38 BW to 3.46 BW for resultant joint forces, while model estimated resultant joint force reaches up 3.6 BW [28, 29, 34, 39].

Lunges and squats both require high knee flexion and moderate to high flexion/extension velocities. Lunges showed the highest anterior, lateral and distal force, while squats showed comparatively high anterior and lateral force and moderate distal force. Previous studies report slightly lower axial knee joint forces of up to 4.6 BW during squats and up to 5.8 BW during lunges [42, 50, 51, 53]. Lunges and squats require the highest quadriceps activity out of all tasks while simultaneously requiring only moderate hamstring activity, which is consistent with EMG measurements [51, 52].

Subsequently, due to their high requirements, especially towards axial knee joint loading and quadriceps activity, lunges and squats should be employed during later rehabilitation phases. At the same time successfully performing these tasks might be good indicator for functional recovery.

Taken together, the results of this study can provide the first step towards a biomechanically informed basis that could help design more personalized rehabilitation programs, especially when combined with current trends towards sensor-based rehabilitation. Wearable sensors have seen growing use in assessing patient outcomes, as they provide objective data on functional outcomes that can benefit patients [65, 66]. For example, Yeung and colleagues found by using an inertial measurement unit fixed below the knee that even though the self-reported measures improved for their entire cohort, some patients did present with a decrease in knee loading, suggesting a decline in functional performance [65]. By combining continuous measurement with insights into internal loading, a personalized rehabilitation program with progressive loading can be developed.

This study has several limitations: First, the study cohort consisted of young and healthy adults. In patients altered kinematics were shown and in older patients additional effects of neuromuscular aging might be present [18–22, 67]. Also, the recruitment algorithm in the musculoskeletal simulation aims to minimize muscle activity. In patients, different underlying strategies like minimizing pain might be present. Further research should examine a meaningful sample of patients to complement the results of the current study.

Second, joint forces and muscle activity were estimated using a musculoskeletal model. While the model has not been directly validated for tasks involving high knee flexion, it has shown good agreement with instrumented implant data [60] and has been used in previous studies reporting similar peak knee flexion (e.g., [68–70]). While marker-less systems are less accurate than marker-based systems, previous studies have demonstrated good agreement with marker-based systems, regarding the simulation software used in the current study [57]. However, as the model does not account for effects such as muscular pretension, joint compression may be underestimated and distraction overestimated. Also, employing both measured and predicted GRF as simulation inputs across different tasks makes it more difficult to interpret task-related differences.

Third, the tasks are only characterized by the maximum value of the selected parameters. No information about integral joint work or the duration of the load is considered. Furthermore, the combination of these parameters e.g. considering at which knee joint angle the maximum force or maximum muscle activity is experienced might add valuable information. Still, the approach of this study adds valuable insights, since the presented maximum values define a lower bound of requirements that help rank the tasks and thereby allow for a progressive increase in difficulty.

Finally, the applied statistical methods must be considered when interpreting the results of this study. Comparing adjacent tasks shows where significant steps between exercises occur but does not compare progressions that might be employed by therapists (e.g. StandUp_S → StandUp → Squat). Also, the lack of significant differences within one level (or in case of hamstring activity within all tasks) does not necessarily mean that there are no significant differences between tasks, but rather that no single step between two adjacent tasks reached significance. The supplementary material contains pair wise comparisons between all tasks that allow a comparison between any two tasks. Additionally, many of the observed differences – even if significant – are relatively small, raising concerns about their clinical relevance. Observed differences in knee kinematics must also be interpreted in the context of the accuracy of the marker-less motion capture system [57]. Furthermore, the absence of a defined minimum clinically relevant difference makes it challenging to determine the practical relevance of these findings.

# Conclusion

This study provides insights into biomechanical requirements associated with selected exercises and ADLs. Differences were observed between tasks, with comparatively high requirements during lunges and squats, medium requirements during sitting down and rising from a chair and low requirements during balance shifts and variations of the single leg stand. ADLs like gait and stair walking show surprisingly high requirements compared to many exercises. These findings are a step towards biomechanically informed exercise selection and the development of personalized rehabilitation programs.

# Supplementary material

## Supplementary material A

Supplementary material A contains images showing all parameters for each task, visualizing the combined demands of the task. The transparent grey bars show the requirements of gait as physiological reference values. Values are reported as median and interquartile range.

## Supplementary material B

Supplementary material B contains images showing pair wise comparisons between all tasks that show significant differences between any two tasks. Significant differences between tasks are shown using the Wilcoxon signed-rank test and Bonferroni correction (n = 190 comparisons).

## Supplementary material C

Supplementary material C contains videos visualizing the progression of the knee joint motion, forces and muscle activity over one movement cycle. Data are taken from the average subject. Note that for visualization purposes the entire motion is only imposed on the femur.

References

1. Louw QA, Manilall J and Grimmer KA. Epidemiology of knee injuries among adolescents: a systematic review. *Br J Sports Med* 2008; 42: 2–10.

2. Heidari B. Knee osteoarthritis prevalence, risk factors, pathogenesis and features: Part I. *Caspian J Intern Med* 2011; 2: 205–212.

3. Wallace IJ, Worthington S, Felson DT, et al. Knee osteoarthritis has doubled in prevalence since the mid-20th century. *Proc Natl Acad Sci U S A* 2017; 114: 9332–9336.

4. Turkiewicz A, Petersson IF, Björk J, et al. Current and future impact of osteoarthritis on health care: a population-based study with projections to year 2032. *Osteoarthritis Cartilage* 2014; 22: 1826–1832.

5. Maradit Kremers H, Larson DR, Crowson CS, et al. Prevalence of Total Hip and Knee Replacement in the United States. *J Bone Joint Surg Am* 2015; 97: 1386–1397.

6. Inacio MCS, Graves SE, Pratt NL, et al. Increase in Total Joint Arthroplasty Projected from 2014 to 2046 in Australia: A Conservative Local Model With International Implications. *Clin Orthop Relat Res* 2017; 475: 2130–2137.

7. Culliford D, Maskell J, Judge A, et al. Future projections of total hip and knee arthroplasty in the UK: results from the UK Clinical Practice Research Datalink. *Osteoarthritis Cartilage* 2015; 23: 594–600.

8. Jüni P, Reichenbach S and Dieppe P. Osteoarthritis: rational approach to treating the individual. *Best Pract Res Clin Rheumatol* 2006; 20: 721–740.

9. Minns Lowe CJ, Barker KL, Dewey M, et al. Effectiveness of physiotherapy exercise after knee arthroplasty for osteoarthritis: systematic review and meta-analysis of randomised controlled trials. *BMJ* 2007; 335: 812, https://www.bmj.com/content/335/7624/812.full (2007).

10. Artz N, Elvers KT, Lowe CM, et al. Effectiveness of physiotherapy exercise following total knee replacement: systematic review and meta-analysis. *BMC Musculoskelet Disord* 2015; 16: 15.

11. Henderson KG, Wallis JA and Snowdon DA. Active physiotherapy interventions following total knee arthroplasty in the hospital and inpatient rehabilitation settings: a systematic review and meta-analysis. *Physiotherapy* 2018; 104: 25–35.

12. Konnyu KJ, Thoma LM, Cao W, et al. Rehabilitation for Total Knee Arthroplasty: A Systematic Review. *Am J Phys Med Rehabil* 2023; 102: 19–33.

13. Jenkins SM, Guzman A, Gardner BB, et al. Rehabilitation After Anterior Cruciate Ligament Injury: Review of Current Literature and Recommendations. *Curr Rev Musculoskelet Med* 2022; 15: 170–179.

14. Arna Risberg M, Lewek M and Snyder-Mackler L. A systematic review of evidence for anterior cruciate ligament rehabilitation: how much and what type? *Physical Therapy in Sport* 2004; 5: 125–145.

15. Logerstedt DS, Ebert JR, MacLeod TD, et al. Effects of and Response to Mechanical Loading on the Knee. *Sports Med* 2022; 52: 201–235.

16. Lange T, Schmitt J, Kopkow C, et al. What Do Patients Expect From Total Knee Arthroplasty? A Delphi Consensus Study on Patient Treatment Goals. *J Arthroplasty* 2017; 32: 2093-2099.e1.

17. Wunderlich F, Eckhard L, Büttner M, et al. The INDICATE Knee expectations survey detects general patient treatment goals for total knee arthroplasty and the influence of demographic factors on patients expectations. *Knee Surg Sports Traumatol Arthrosc* 2023; 31: 892–904.

18. Gaffney BM, Harris MD, Davidson BS, et al. Multi-Joint Compensatory Effects of Unilateral Total Knee Arthroplasty During High-Demand Tasks. *Ann Biomed Eng* 2016; 44: 2529–2541.

19. McClelland JA, Feller JA, Menz HB, et al. Patients with total knee arthroplasty do not use all of their available range of knee flexion during functional activities. *Clin Biomech (Bristol, Avon)* 2017; 43: 74–78.

20. Vroey H de, Staes F, Vereecke E, et al. Lower extremity gait kinematics outcomes after knee replacement demonstrate arthroplasty-specific differences between unicondylar and total knee arthroplasty: A pilot study. *Gait Posture* 2019; 73: 299–304.

21. Kong L, Lai H, Zeng X, et al. The evolution of three-dimensional knee kinematics after ACL reconstruction within one year. *Front. Bioeng. Biotechnol.* 2025; 13.

22. Dai B, Butler RJ, Garrett WE, et al. Anterior cruciate ligament reconstruction in adolescent patients: limb asymmetry and functional knee bracing. *Am J Sports Med* 2012; 40: 2756–2763.

23. Farquhar SJ, Reisman DS and Snyder-Mackler L. Persistence of altered movement patterns during a sit-to-stand task 1 year following unilateral total knee arthroplasty. *Physical Therapy* 2008; 88: 567–579.

24. Mizner RL and Snyder-Mackler L. Altered loading during walking and sit-to-stand is affected by quadriceps weakness after total knee arthroplasty. *J Orthop Res* 2005; 23: 1083–1090.

25. Vroey H de, Staes F, Weygers I, et al. Hip and knee kinematics of the forward lunge one year after unicondylar and total knee arthroplasty. *J Electromyogr Kinesiol* 2019; 48: 24–30.

26. Tayfur B, Charuphongsa C, Morrissey D, et al. Neuromuscular Function of the Knee Joint Following Knee Injuries: Does It Ever Get Back to Normal? A Systematic Review with Meta-Analyses. *Sports Med* 2021; 51: 321–338.

27. Petterson SC, Mizner RL, Stevens JE, et al. Improved function from progressive strengthening interventions after total knee arthroplasty: a randomized clinical trial with an imbedded prospective cohort. *Arthritis Rheum* 2009; 61: 174–183.

28. Kutzner I, Heinlein B, Graichen F, et al. Loading of the knee joint during activities of daily living measured in vivo in five subjects. *J Biomech* 2010; 43: 2164–2173.

29. Heinlein B, Kutzner I, Graichen F, et al. ESB Clinical Biomechanics Award 2008: Complete data of total knee replacement loading for level walking and stair climbing measured in vivo with a follow-up of 6-10 months. *Clin Biomech (Bristol, Avon)* 2009; 24: 315–326.

30. Obrębska P, Skubich J and Piszczatowski S. Gender differences in the knee joint loadings during gait. *Gait Posture* 2020; 79: 195–202.

31. Chen TL-W, Wong DW-C, Xu Z, et al. Lower limb muscle co-contraction and joint loading of flip-flops walking in male wearers. *PLoS One* 2018; 13: e0193653.

32. D'Lima DD, Patil S, Steklov N, et al. Tibial forces measured in vivo after total knee arthroplasty. *J Arthroplasty* 2006; 21: 255–262.

33. Taylor WR, Heller MO, Bergmann G, et al. Tibio-femoral loading during human gait and stair climbing. *J Orthop Res* 2004; 22: 625–632.

34. Taylor WR, Schütz P, Bergmann G, et al. A comprehensive assessment of the musculoskeletal system: The CAMS-Knee data set. *J Biomech* 2017; 65: 32–39.

35. Choi BS, Kwon SB, Jeon S, et al. Relationship between muscle activation and sagittal knee joint biomechanics in patients with patellofemoral pain syndrome: a cross-sectional study. *Knee Surg Relat Res* 2025; 37: 7.

36. Liikavainio T, Isolehto J, Helminen HJ, et al. Loading and gait symmetry during level and stair walking in asymptomatic subjects with knee osteoarthritis: importance of quadriceps femoris in reducing impact force during heel strike? *Knee* 2007; 14: 231–238.

37. Hyodo K, Kanamori A, Kadone H, et al. Gait Analysis Comparing Kinematic, Kinetic, and Muscle Activation Data of Modern and Conventional Total Knee Arthroplasty. *Arthroplast Today* 2020; 6: 338–342.

38. Burden AM, Trew M and Baltzopoulos V. Normalisation of gait EMGs: a re-examination. *J Electromyogr Kinesiol* 2003; 13: 519–532.

39. van Rossom S, Smith CR, Thelen DG, et al. Knee Joint Loading in Healthy Adults During Functional Exercises: Implications for Rehabilitation Guidelines. *J Orthop Sports Phys Ther* 2018; 48: 162–173.

40. Trepczynski A, Kutzner I, Kornaropoulos E, et al. Patellofemoral joint contact forces during activities with high knee flexion. *J Orthop Res* 2012; 30: 408–415.

41. Comfort P, Jones PA, Smith LC, et al. Joint Kinetics and Kinematics During Common Lower Limb Rehabilitation Exercises. *J Athl Train* 2015; 50: 1011–1018.

42. Kang K-T, Koh Y-G, Jung M, et al. The effects of posterior cruciate ligament deficiency on posterolateral corner structures under gait- and squat-loading conditions: A computational knee model. *Bone Joint Res* 2017; 6: 31–42.

43. Rasnick R, Standifird T, Reinbolt JA, et al. Knee Joint Loads and Surrounding Muscle Forces during Stair Ascent in Patients with Total Knee Replacement. *PLoS One* 2016; 11: e0156282.

44. Hortobágyi T, Westerkamp L, Beam S, et al. Altered hamstring-quadriceps muscle balance in patients with knee osteoarthritis. *Clin Biomech (Bristol, Avon)* 2005; 20: 97–104.

45. Marshall RN, Morgan PT, Martinez-Valdes E, et al. Quadriceps muscle electromyography activity during physical activities and resistance exercise modes in younger and older adults. *Exp Gerontol* 2020; 136: 110965.

46. Hammond CA, Hatfield GL, Gilbart MK, et al. Trunk and lower limb biomechanics during stair climbing in people with and without symptomatic femoroacetabular impingement. *Clin Biomech (Bristol, Avon)* 2017; 42: 108–114.

47. Bjerke J, Öhberg F, Nilsson KG, et al. Compensatory strategies for muscle weakness during stair ascent in subjects with total knee arthroplasty. *J Arthroplasty* 2014; 29: 1499–1502.

48. Petrella M, Selistre LFA, Serrão PRMS, et al. Kinetics, kinematics, and knee muscle activation during sit to stand transition in unilateral and bilateral knee osteoarthritis. *Gait Posture* 2021; 86: 38–44.

49. Kowalski E, Pelegrinelli ARM, Catelli DS, et al. Medial and lateral knee contact forces and muscle forces during sit-to-stand in patients one year after unilateral total knee arthroplasty. *Med Eng Phys* 2024; 134: 104262.

50. Lu Y, Mei Q, Peng H-T, et al. A Comparative Study on Loadings of the Lower Extremity during Deep Squat in Asian and Caucasian Individuals via OpenSim Musculoskeletal Modelling. *BioMed Research International* 2020; 2020.

51. Kothurkar R, Lekurwale R, Gad M, et al. Estimation and Comparison of Knee Joint Contact Forces During Heel Contact and Heel Rise Deep Squatting. *Indian J Orthop* 2023; 57: 310–318.

52. Park S, Huang T, Song J, et al. Comparative Study of the Biomechanical Factors in Range of Motion, Muscle Activity, and Vertical Ground Reaction Force between a Forward Lunge and Backward Lunge. *PTRS* 2021; 10: 98–105.

53. Dejtiar DL, Dzialo CM, Pedersen PH, et al. Development and Evaluation of a Subject-Specific Lower Limb Model With an Eleven-Degrees-of-Freedom Natural Knee Model Using Magnetic Resonance and Biplanar X-Ray Imaging During a Quasi-Static Lunge. *J Biomech Eng* 2020; 142.

54. Donath L, Kurz E, Roth R, et al. Leg and trunk muscle coordination and postural sway during increasingly difficult standing balance tasks in young and older adults. *Maturitas* 2016; 91: 60–68.

55. Bencke J, Strøm M, Curtis DJ, et al. Differences in Thigh Muscle Activation Between Standing and Landing Exercises for Knee Injury Prevention and Rehabilitation. *Int J Sports Phys Ther* 2023; 18: 102–112.

56. Fluit R, Andersen MS, Kolk S, et al. Prediction of ground reaction forces and moments during various activities of daily living. *J Biomech* 2014; 47: 2321–2329.

57. Auer S, Süß F and Dendorfer S. Using markerless motion capture and musculoskeletal models: An evaluation of joint kinematics. *Technol Health Care* 2024; 32: 3433–3442.

58. Deutsches Institut für Normung e.V. 18065:2020-08. Gebäudetreppen - Begriffe, Messregeln, Hauptmaße.

59. Bohannon RW. Reference values for the five-repetition sit-to-stand test: a descriptive meta-analysis of data from elders. *Percept Mot Skills* 2006; 103: 215–222.

60. Pieri E de, Lund ME, Gopalakrishnan A, et al. Refining muscle geometry and wrapping in the TLEM 2 model for improved hip contact force prediction. *PLoS One* 2018; 13: e0204109.

61. Rasmussen J, Zee M de, Damsgaard M, et al. A General Method for Scaling Musculo-Skeletal Models. In: *2005 International Symposium on Computer Simulation in Biomechanics,* Cleveland, Ohio, United States.

62. Lund M, Rasmussen J and Andersen M. AnyPyTools: A Python package for reproducible research with the AnyBody Modeling System. *JOSS* 2019; 4: 1108.

63. Dávila Castrodad IM, Recai TM, Abraham MM, et al. Rehabilitation protocols following total knee arthroplasty: a review of study designs and outcome measures. *Ann Transl Med* 2019; 7: S255.

64. Boonstra MC, Schwering PJA, Waal Malefijt MC de, et al. Sit-to-stand movement as a performance-based measure for patients with total knee arthroplasty. *Physical Therapy* 2010; 90: 149–156.

65. Yeung S, Kim HK, Carleton A, et al. Integrating wearables and modelling for monitoring rehabilitation following total knee joint replacement. *Comput Methods Programs Biomed* 2022; 225: 107063.

66. Gianzina E, Kalinterakis G, Delis S, et al. Evaluation of gait recovery after total knee arthroplasty using wearable inertial sensors: A systematic review. *Knee* 2023; 41: 190–203.

67. Clark BC. Neuromuscular Changes with Aging and Sarcopenia. *J Frailty Aging* 2019; 8: 7–9.

68. Roeck J de, van Houcke J, Almeida D, et al. Statistical Modeling of Lower Limb Kinetics During Deep Squat and Forward Lunge. *Front. Bioeng. Biotechnol.* 2020; 8: 233.

69. Takeshita Y, Kiyama R, Kawada M, et al. Effect of hand support during squats on tibiofemoral and patellofemoral joint forces. *J Biomech* 2025; 189: 112814.

70. Chaiyasit K, Phombut C, Wiangkham A, et al. Kinetics prediction of normal knee and undergone total knee arthroplasty during squatting based on extreme gradient boosting. *Results in Engineering* 2024; 23: 102663.
