# Supplementary figures and images for: Comparing kinematic and kinetic demands on the knee joint during selected physiotherapy exercises and activities of daily living
Source: Technol Health Care. 2026 Mar 31;34(3):408–21. doi: 10.1177/09287329251413413 (PMC13195061; doi:10.1177/09287329251413413)

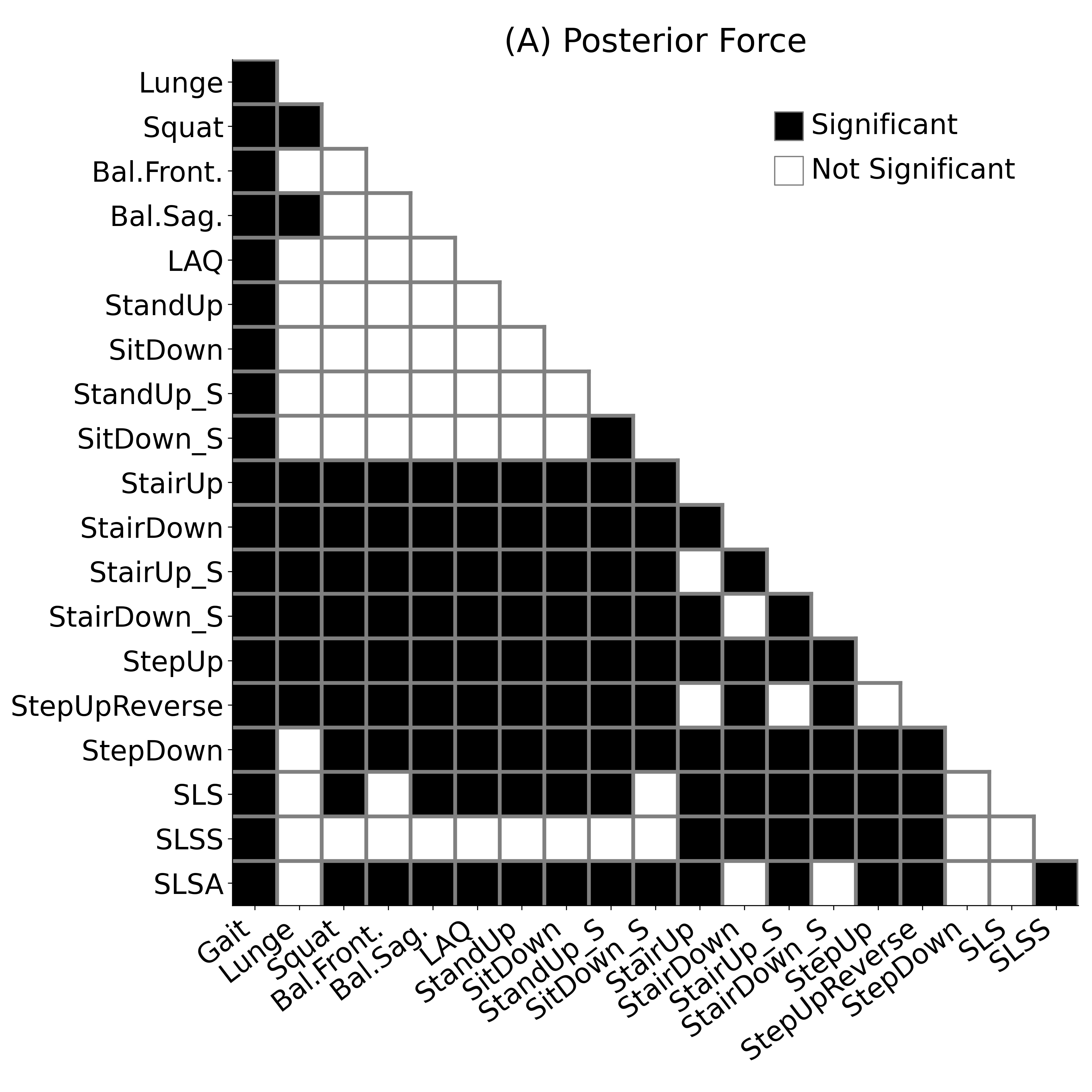

Supplement: sj-zip-1-thc-10.1177_09287329251413413 - Supplemental material for Comparing kinematic and kinetic demands on the knee joint during selected physiotherapy exercises and activities of daily living [file sj-zip-1-thc-10.1177_09287329251413413.zip › Comparison Posterior Force.png]

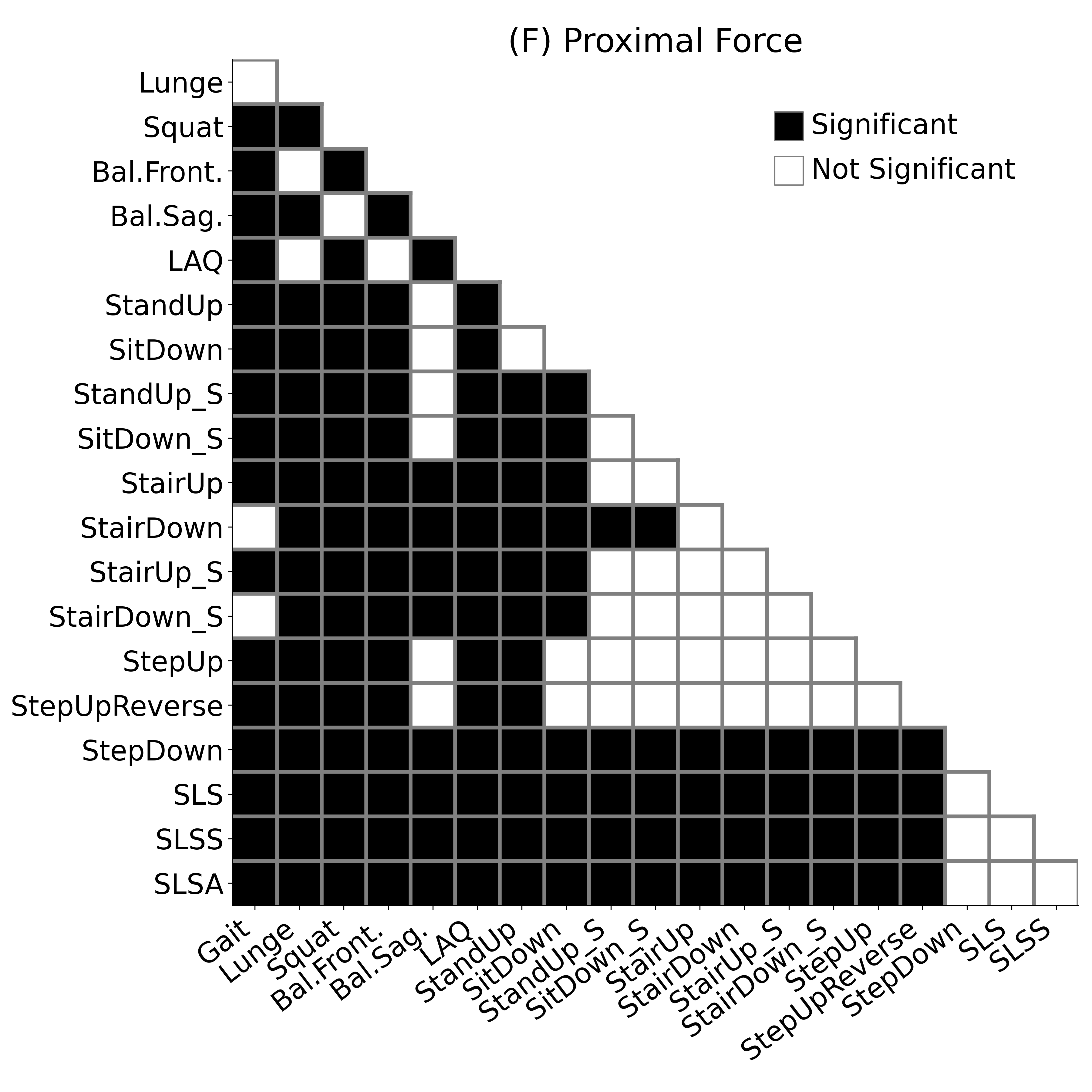

Supplement: sj-zip-1-thc-10.1177_09287329251413413 - Supplemental material for Comparing kinematic and kinetic demands on the knee joint during selected physiotherapy exercises and activities of daily living [file sj-zip-1-thc-10.1177_09287329251413413.zip › Comparison Proximal Force.png]

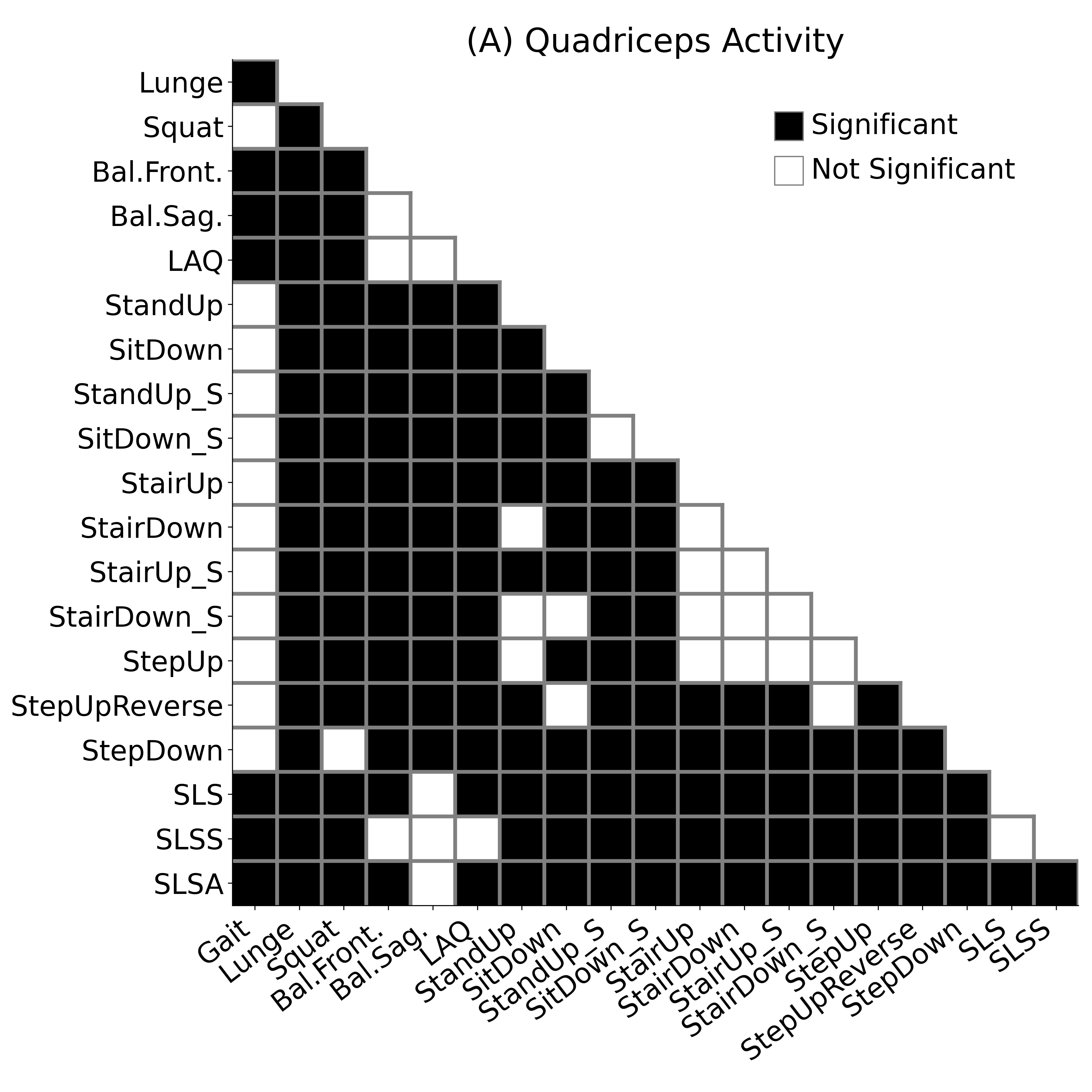

Supplement: sj-zip-1-thc-10.1177_09287329251413413 - Supplemental material for Comparing kinematic and kinetic demands on the knee joint during selected physiotherapy exercises and activities of daily living [file sj-zip-1-thc-10.1177_09287329251413413.zip › Comparison Quadriceps Activity.png]

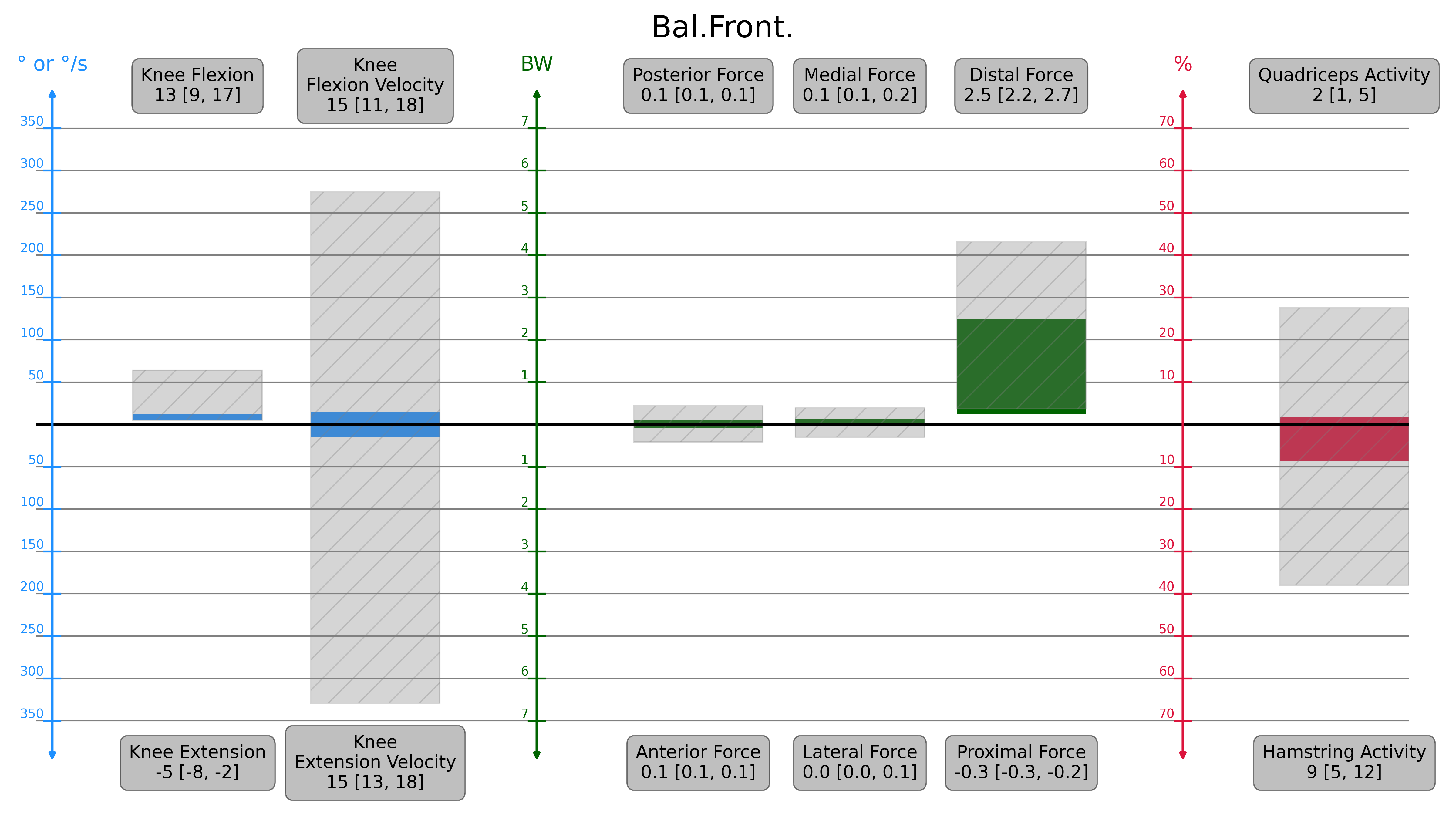

Supplement: sj-zip-1-thc-10.1177_09287329251413413 - Supplemental material for Comparing kinematic and kinetic demands on the knee joint during selected physiotherapy exercises and activities of daily living [file sj-zip-1-thc-10.1177_09287329251413413.zip › Task_Bal.Front..png]

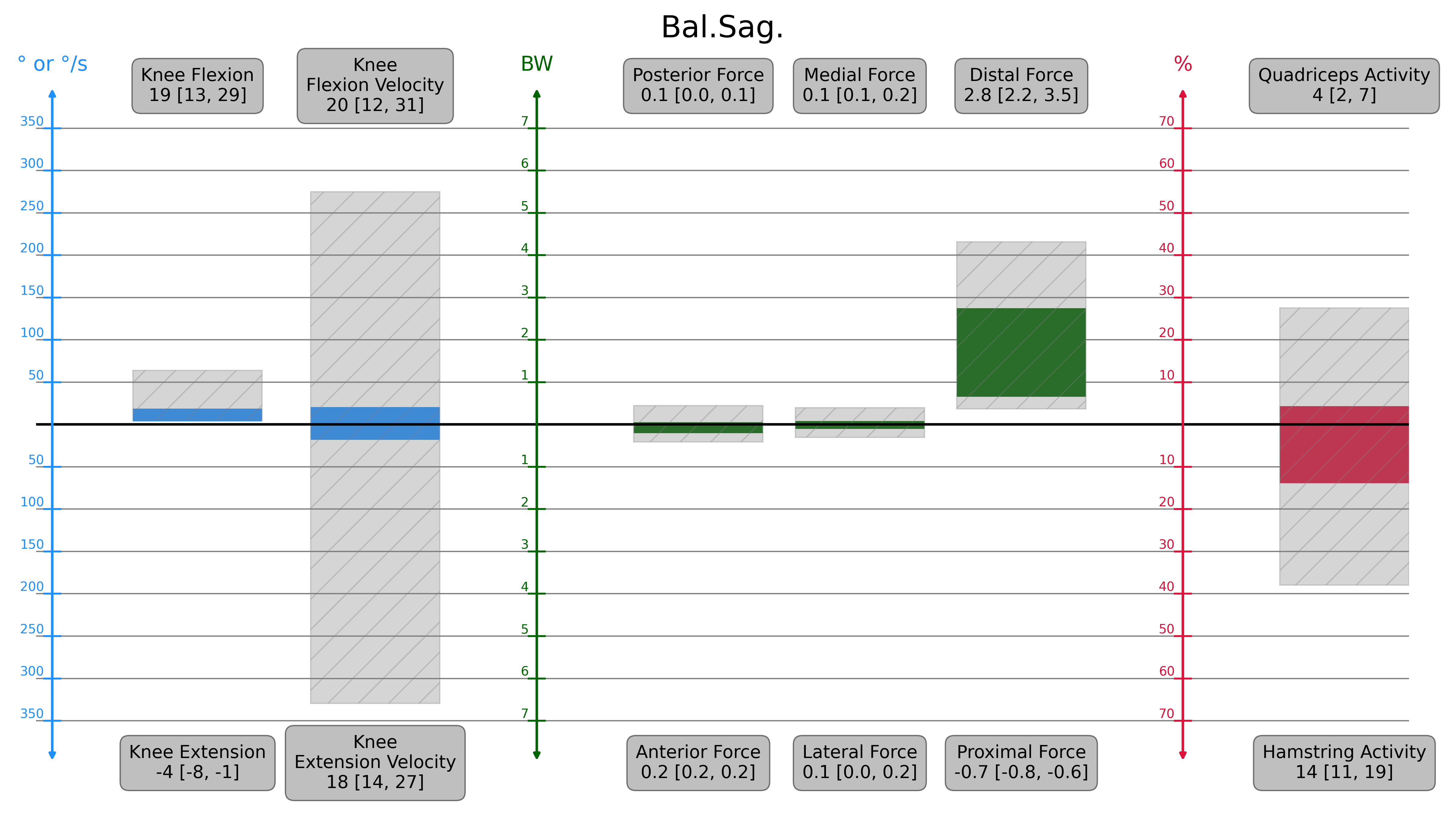

Supplement: sj-zip-1-thc-10.1177_09287329251413413 - Supplemental material for Comparing kinematic and kinetic demands on the knee joint during selected physiotherapy exercises and activities of daily living [file sj-zip-1-thc-10.1177_09287329251413413.zip › Task_Bal.Sag..png]

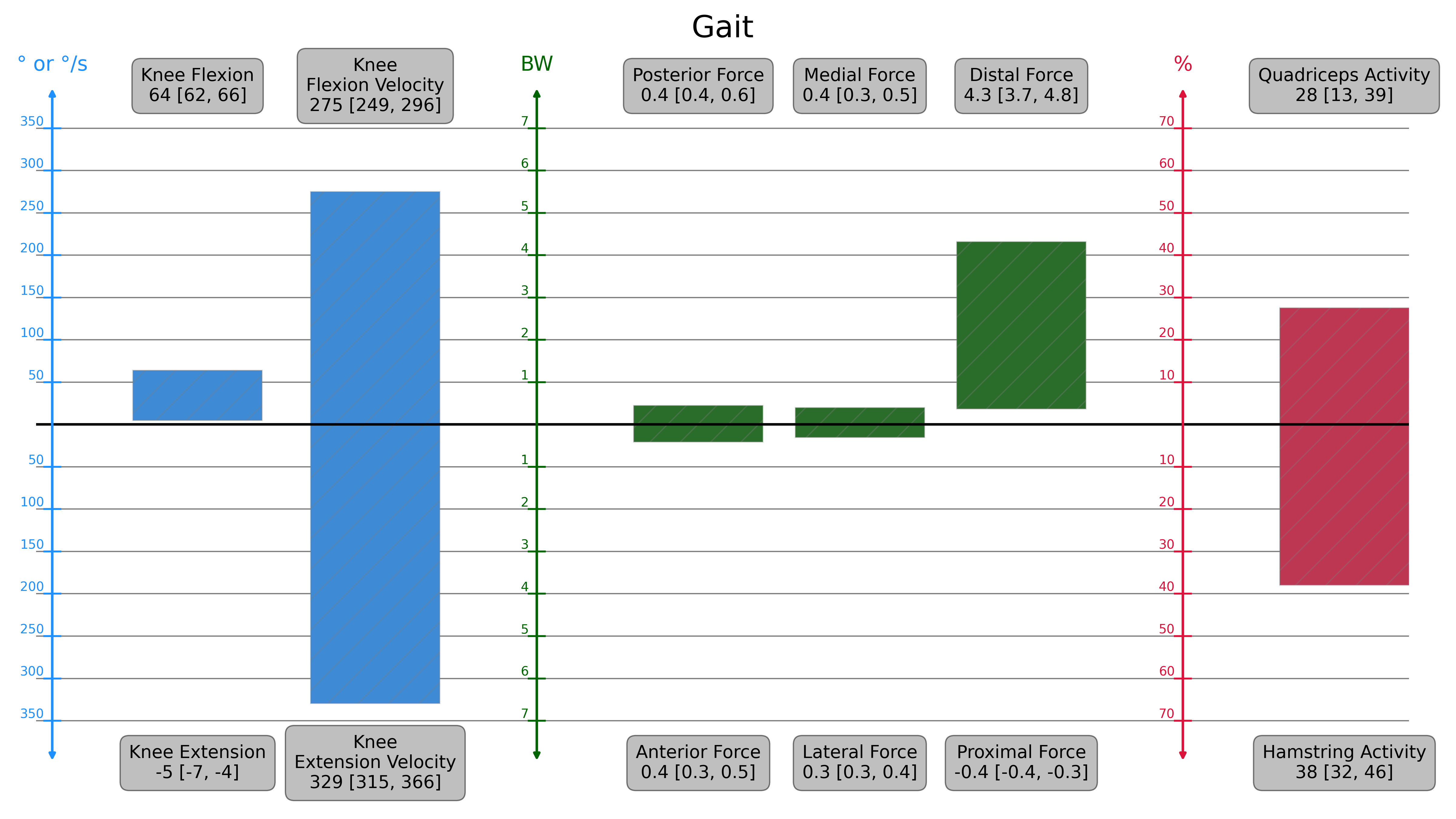

Supplement: sj-zip-1-thc-10.1177_09287329251413413 - Supplemental material for Comparing kinematic and kinetic demands on the knee joint during selected physiotherapy exercises and activities of daily living [file sj-zip-1-thc-10.1177_09287329251413413.zip › Task_Gait.png]

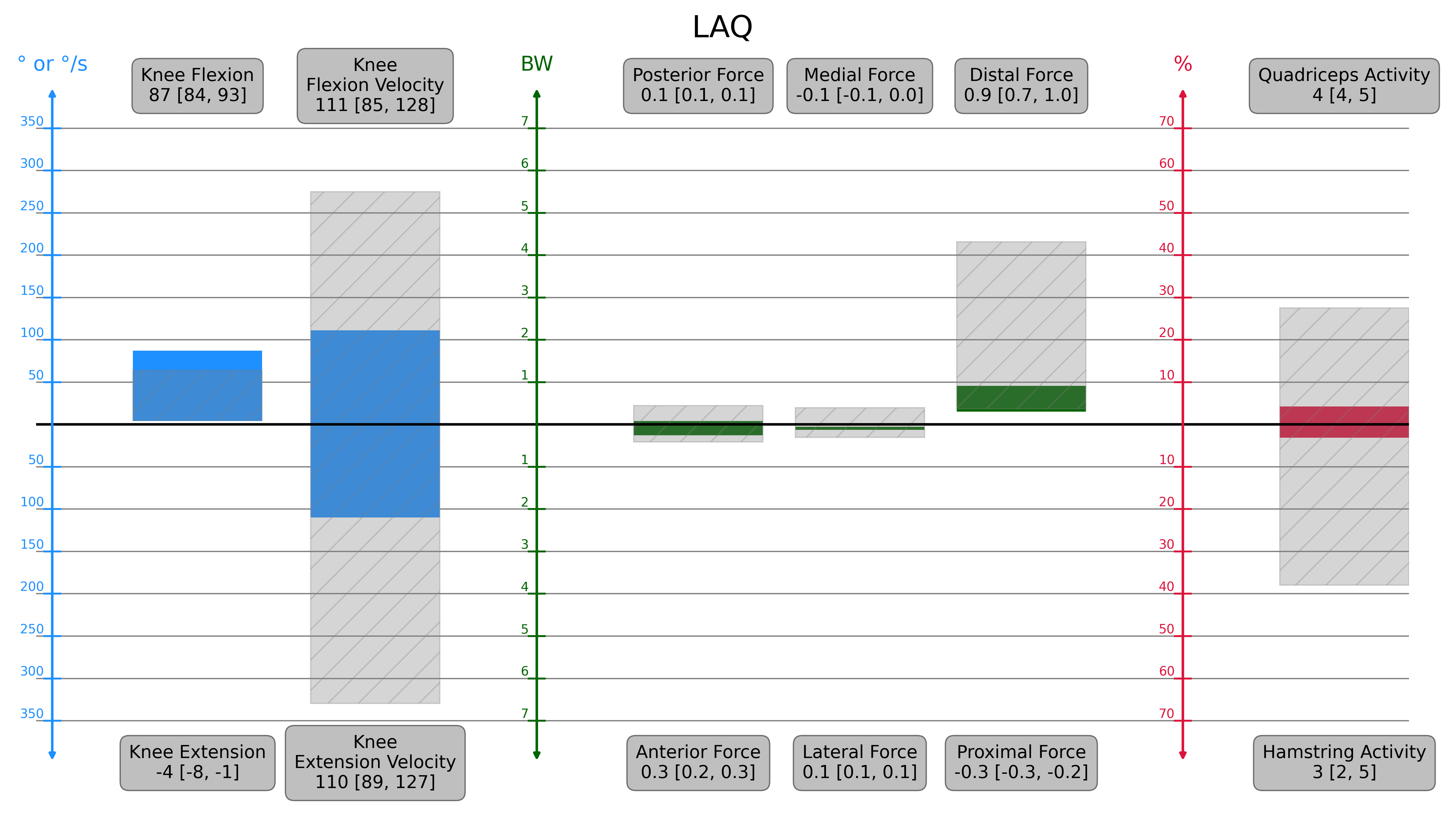

Supplement: sj-zip-1-thc-10.1177_09287329251413413 - Supplemental material for Comparing kinematic and kinetic demands on the knee joint during selected physiotherapy exercises and activities of daily living [file sj-zip-1-thc-10.1177_09287329251413413.zip › Task_LAQ.png]

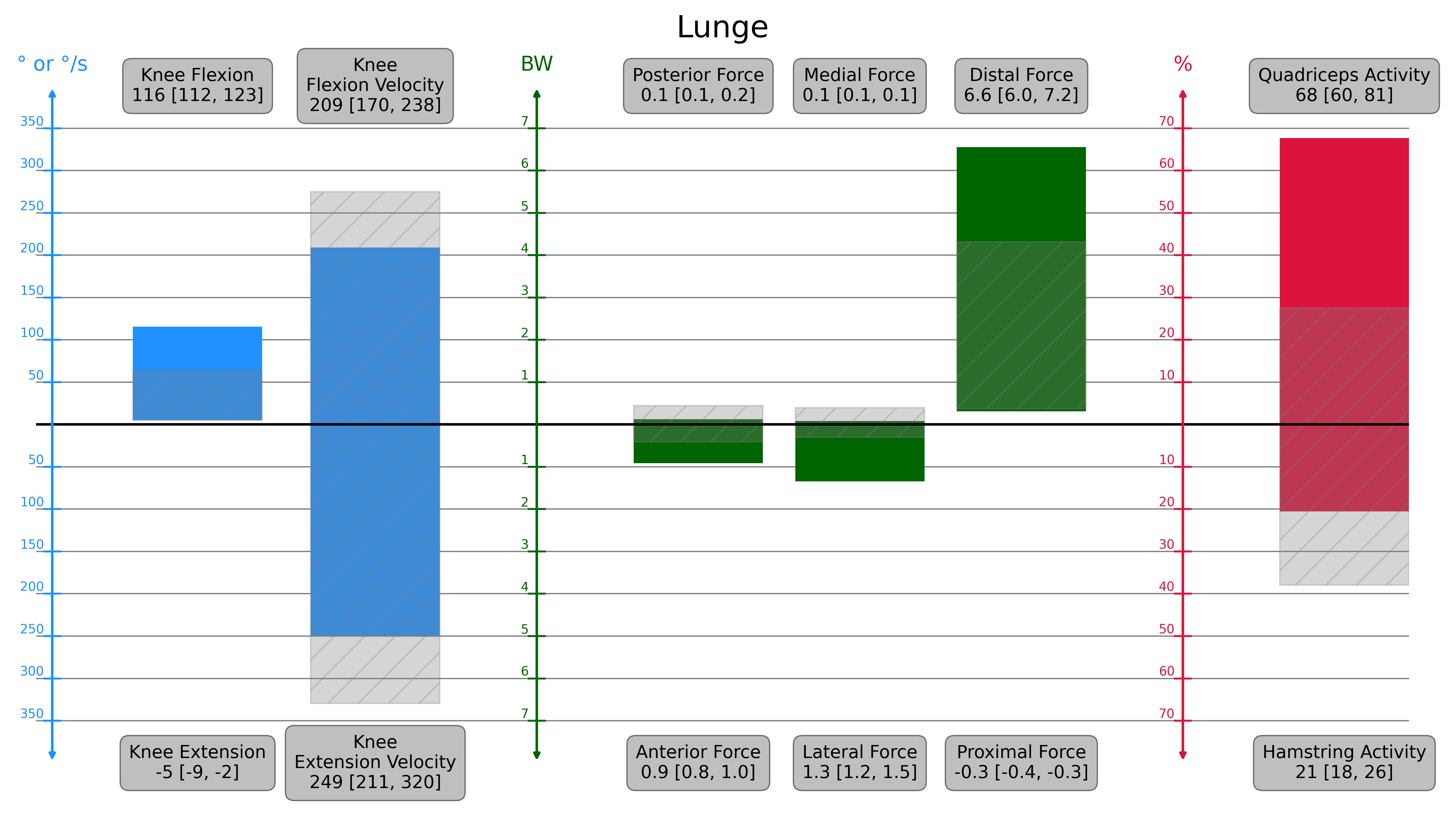

Supplement: sj-zip-1-thc-10.1177_09287329251413413 - Supplemental material for Comparing kinematic and kinetic demands on the knee joint during selected physiotherapy exercises and activities of daily living [file sj-zip-1-thc-10.1177_09287329251413413.zip › Task_Lunge.png]

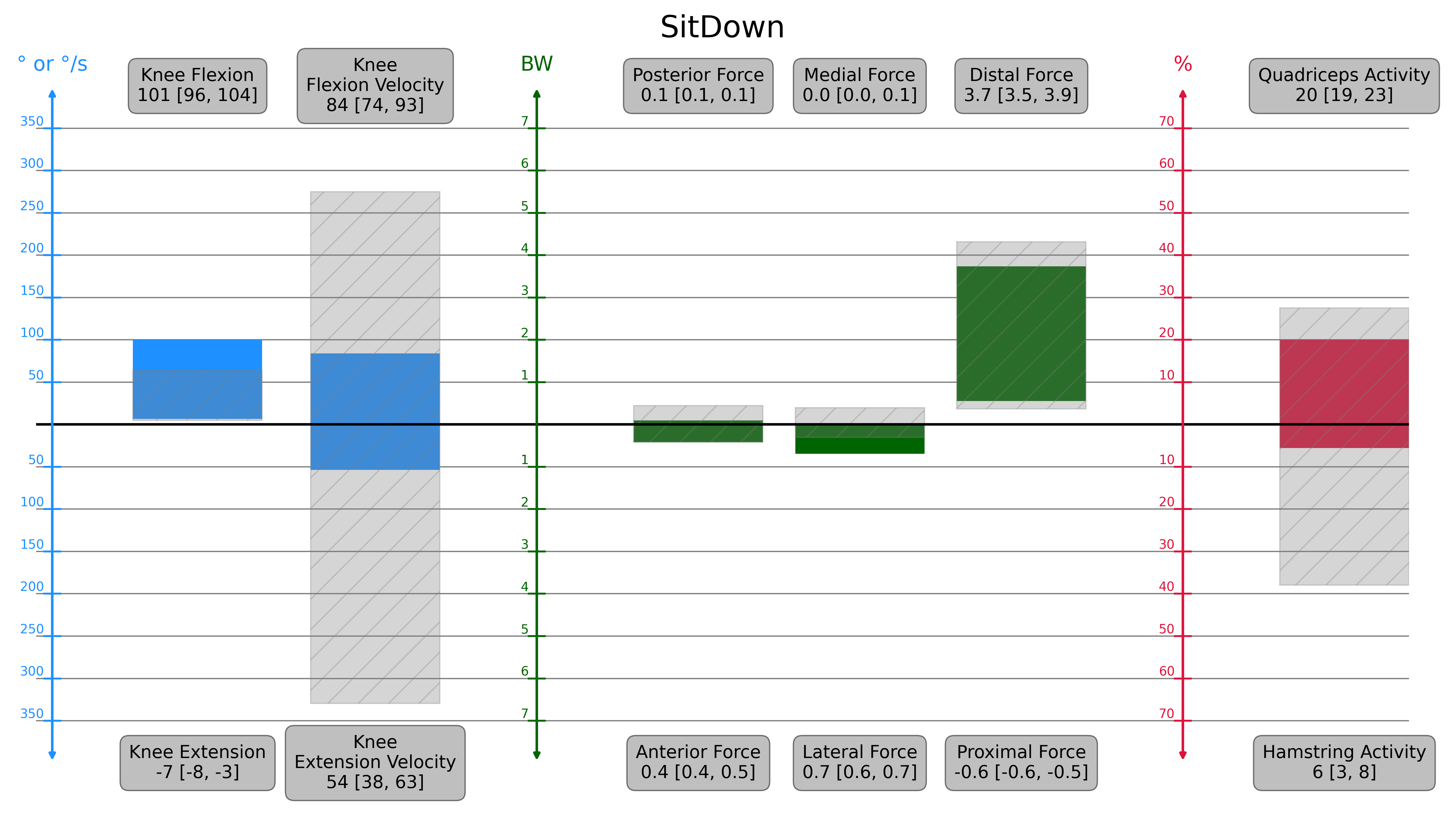

Supplement: sj-zip-1-thc-10.1177_09287329251413413 - Supplemental material for Comparing kinematic and kinetic demands on the knee joint during selected physiotherapy exercises and activities of daily living [file sj-zip-1-thc-10.1177_09287329251413413.zip › Task_SitDown.png]

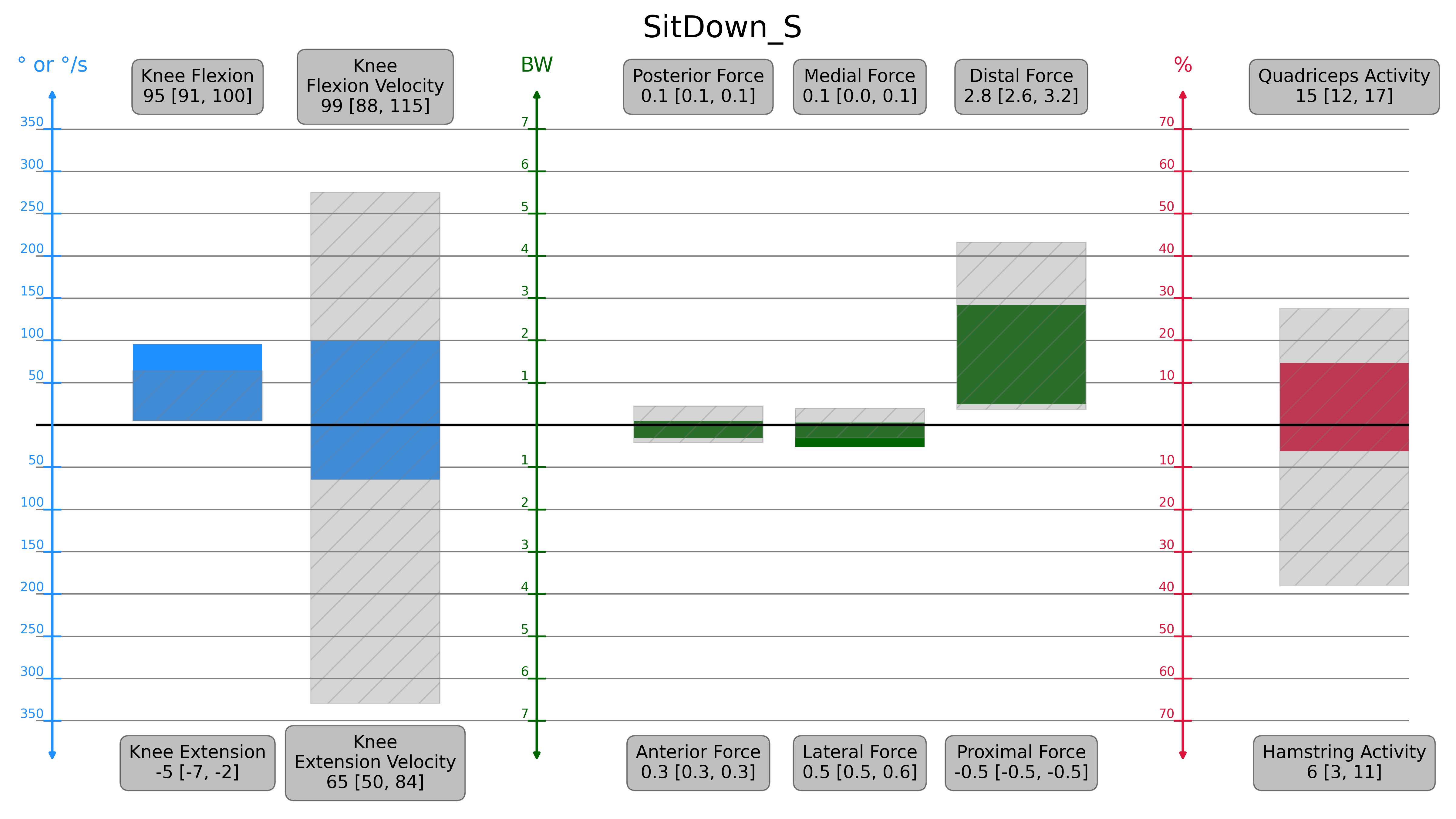

Supplement: sj-zip-1-thc-10.1177_09287329251413413 - Supplemental material for Comparing kinematic and kinetic demands on the knee joint during selected physiotherapy exercises and activities of daily living [file sj-zip-1-thc-10.1177_09287329251413413.zip › Task_SitDown_S.png]

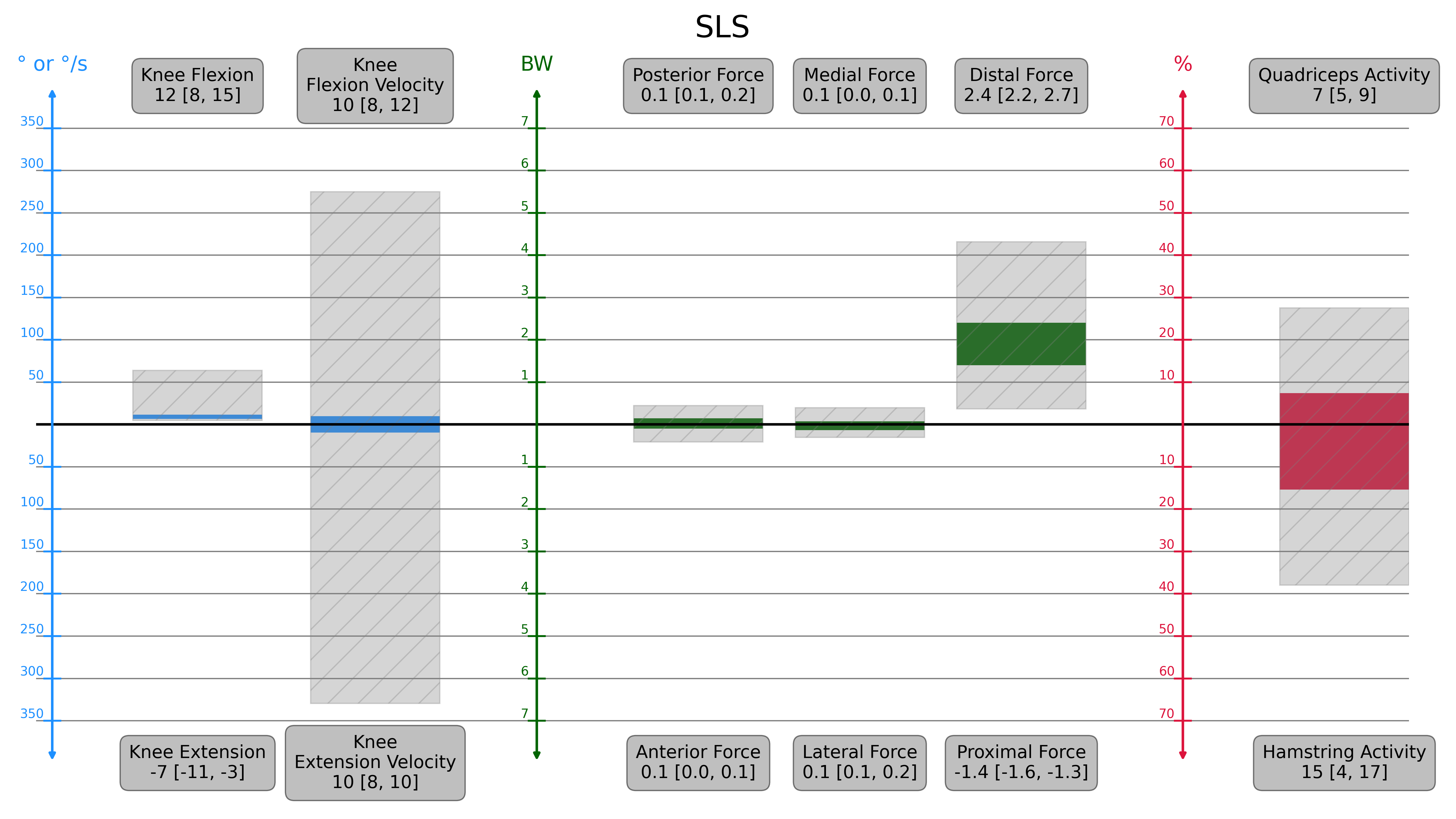

Supplement: sj-zip-1-thc-10.1177_09287329251413413 - Supplemental material for Comparing kinematic and kinetic demands on the knee joint during selected physiotherapy exercises and activities of daily living [file sj-zip-1-thc-10.1177_09287329251413413.zip › Task_SLS.png]

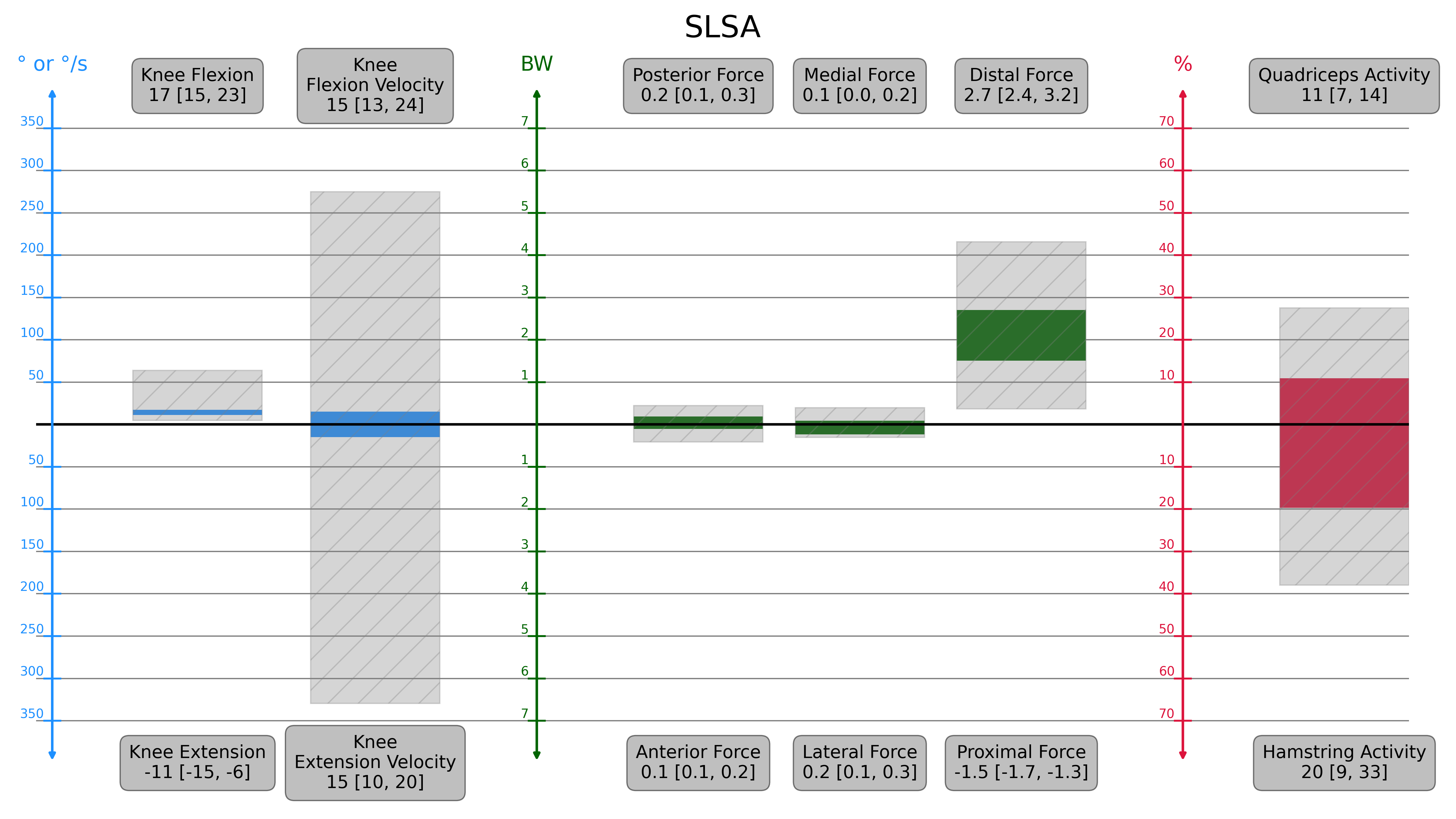

Supplement: sj-zip-1-thc-10.1177_09287329251413413 - Supplemental material for Comparing kinematic and kinetic demands on the knee joint during selected physiotherapy exercises and activities of daily living [file sj-zip-1-thc-10.1177_09287329251413413.zip › Task_SLSA.png]

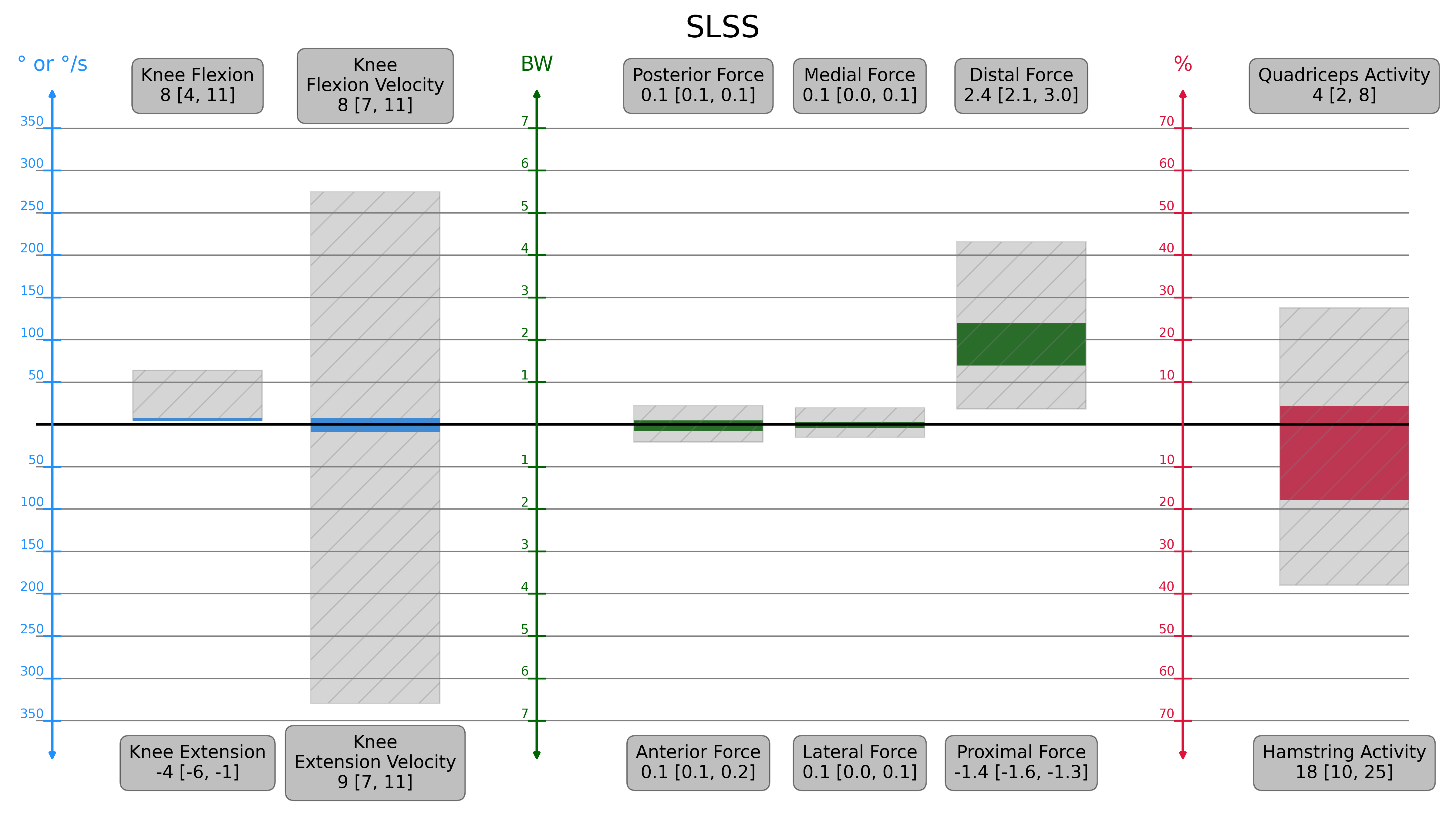

Supplement: sj-zip-1-thc-10.1177_09287329251413413 - Supplemental material for Comparing kinematic and kinetic demands on the knee joint during selected physiotherapy exercises and activities of daily living [file sj-zip-1-thc-10.1177_09287329251413413.zip › Task_SLSS.png]

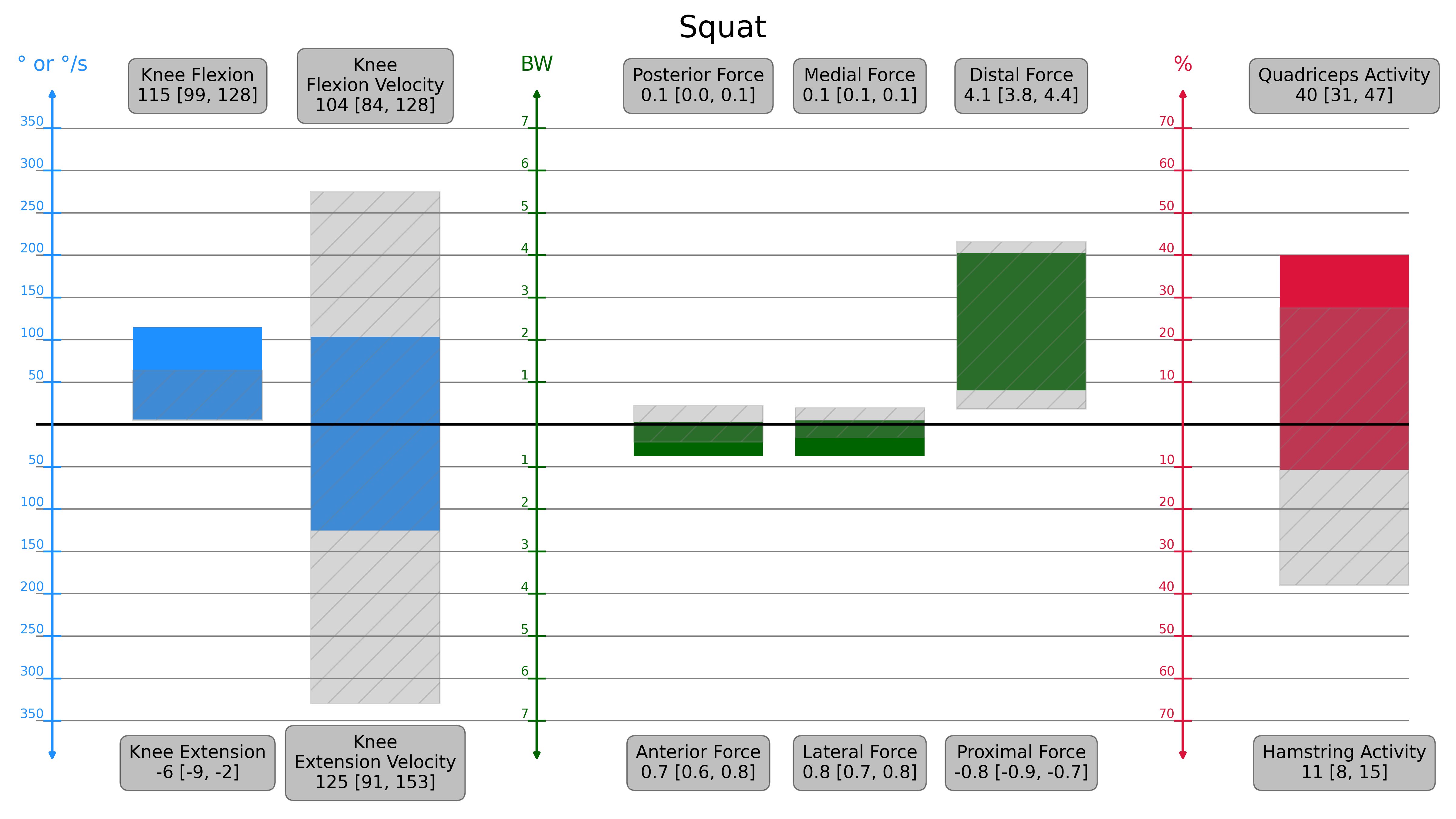

Supplement: sj-zip-1-thc-10.1177_09287329251413413 - Supplemental material for Comparing kinematic and kinetic demands on the knee joint during selected physiotherapy exercises and activities of daily living [file sj-zip-1-thc-10.1177_09287329251413413.zip › Task_Squat.png]

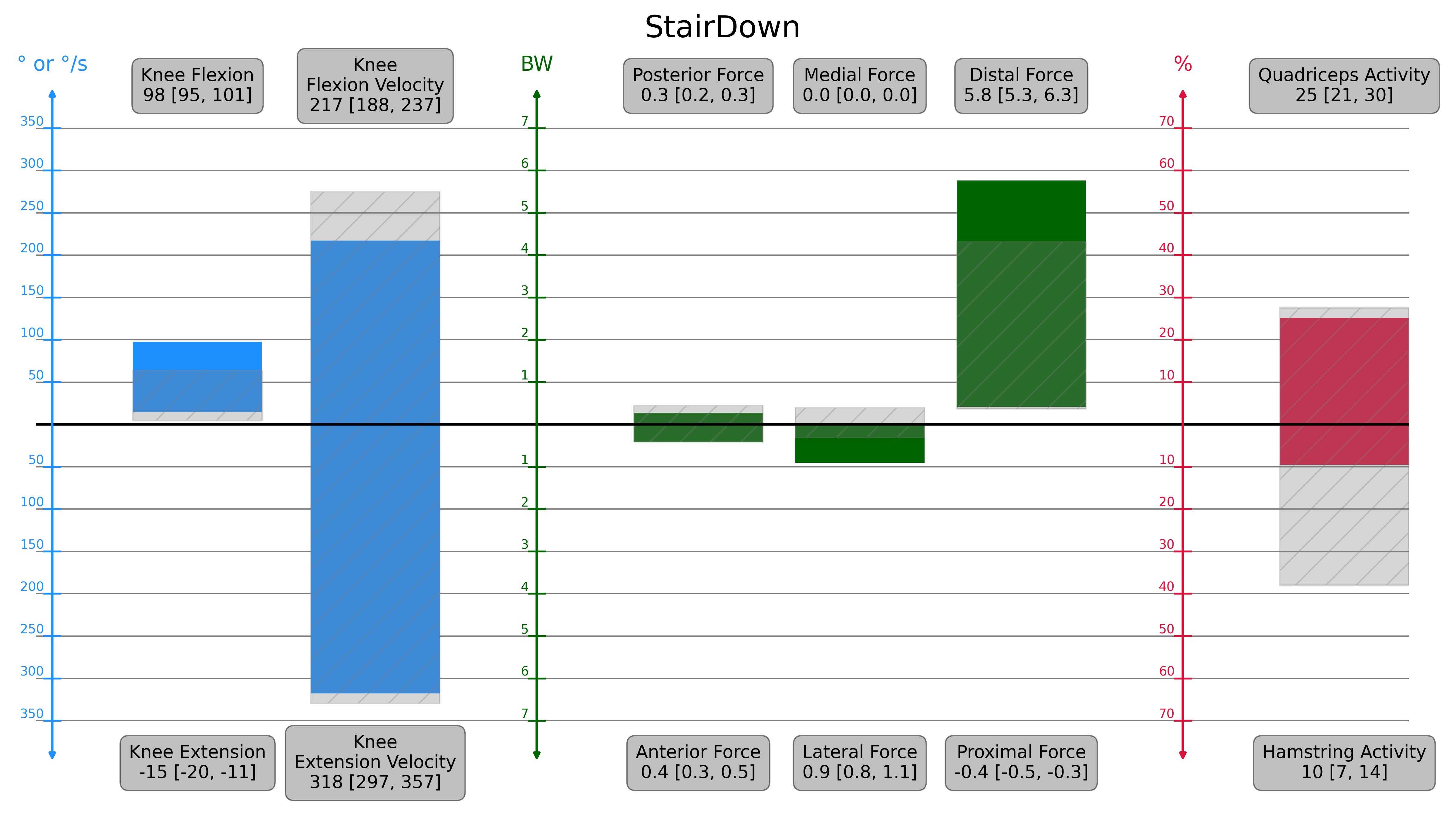

Supplement: sj-zip-1-thc-10.1177_09287329251413413 - Supplemental material for Comparing kinematic and kinetic demands on the knee joint during selected physiotherapy exercises and activities of daily living [file sj-zip-1-thc-10.1177_09287329251413413.zip › Task_StairDown.png]

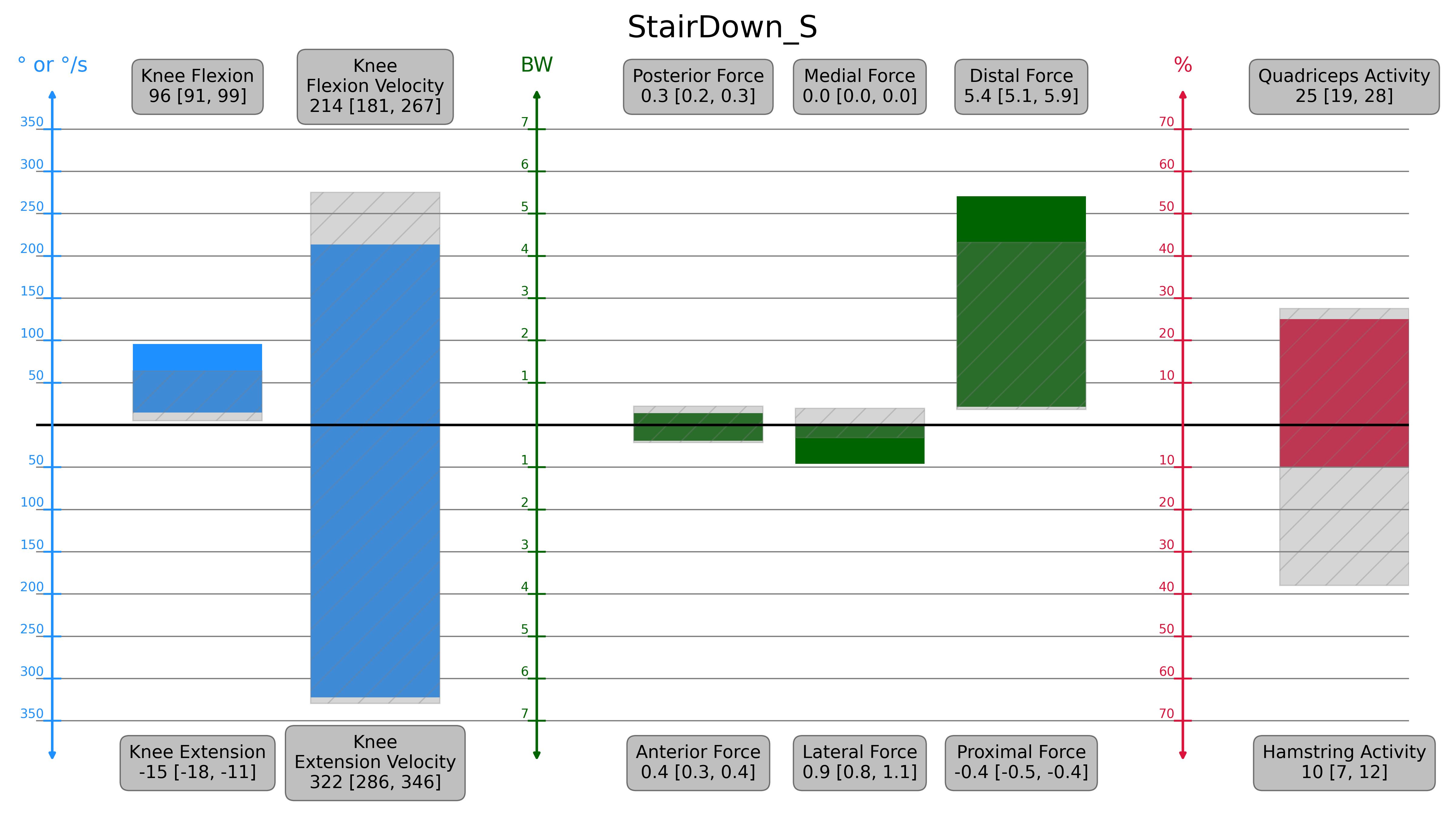

Supplement: sj-zip-1-thc-10.1177_09287329251413413 - Supplemental material for Comparing kinematic and kinetic demands on the knee joint during selected physiotherapy exercises and activities of daily living [file sj-zip-1-thc-10.1177_09287329251413413.zip › Task_StairDown_S.png]

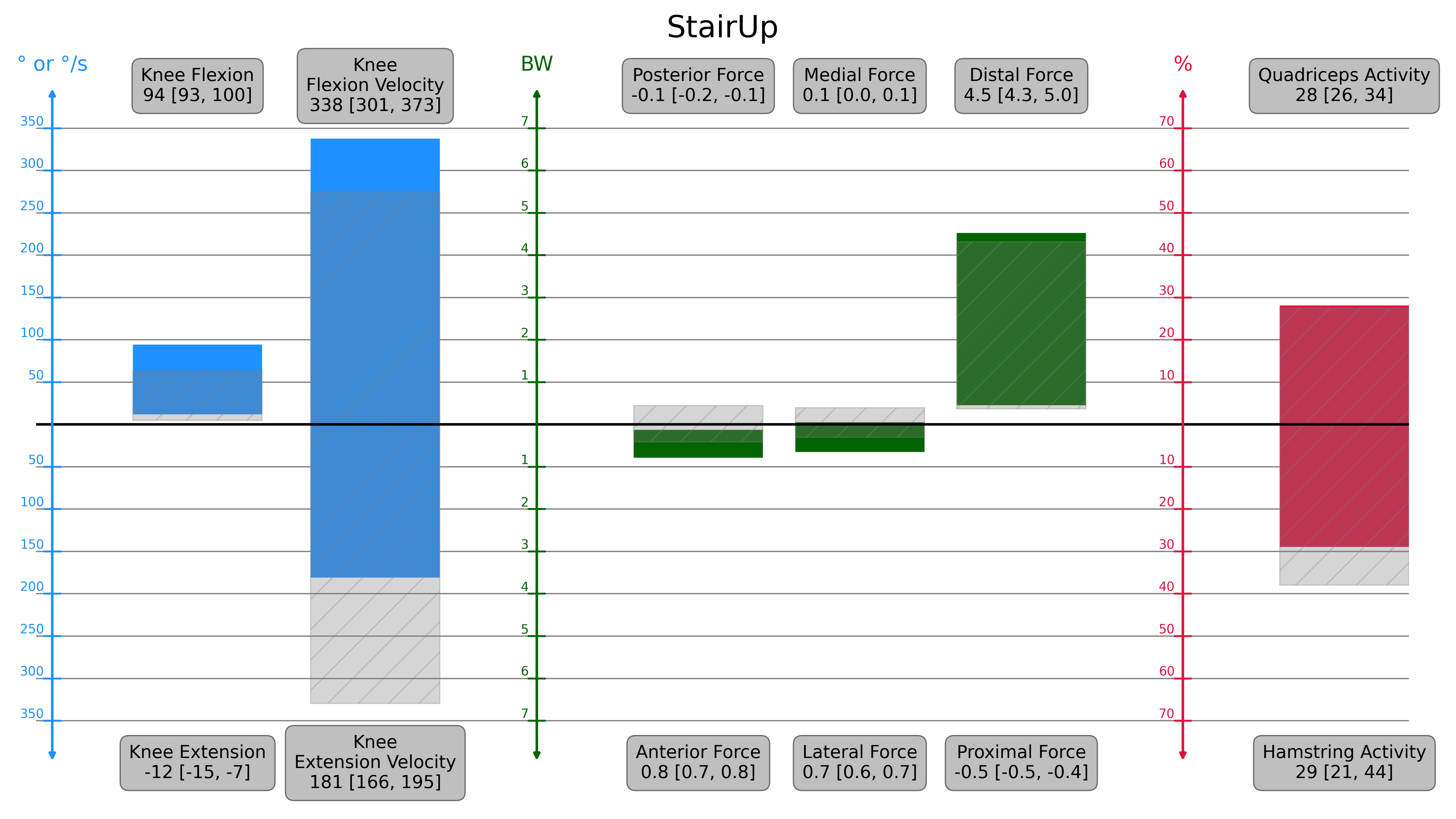

Supplement: sj-zip-1-thc-10.1177_09287329251413413 - Supplemental material for Comparing kinematic and kinetic demands on the knee joint during selected physiotherapy exercises and activities of daily living [file sj-zip-1-thc-10.1177_09287329251413413.zip › Task_StairUp.png]

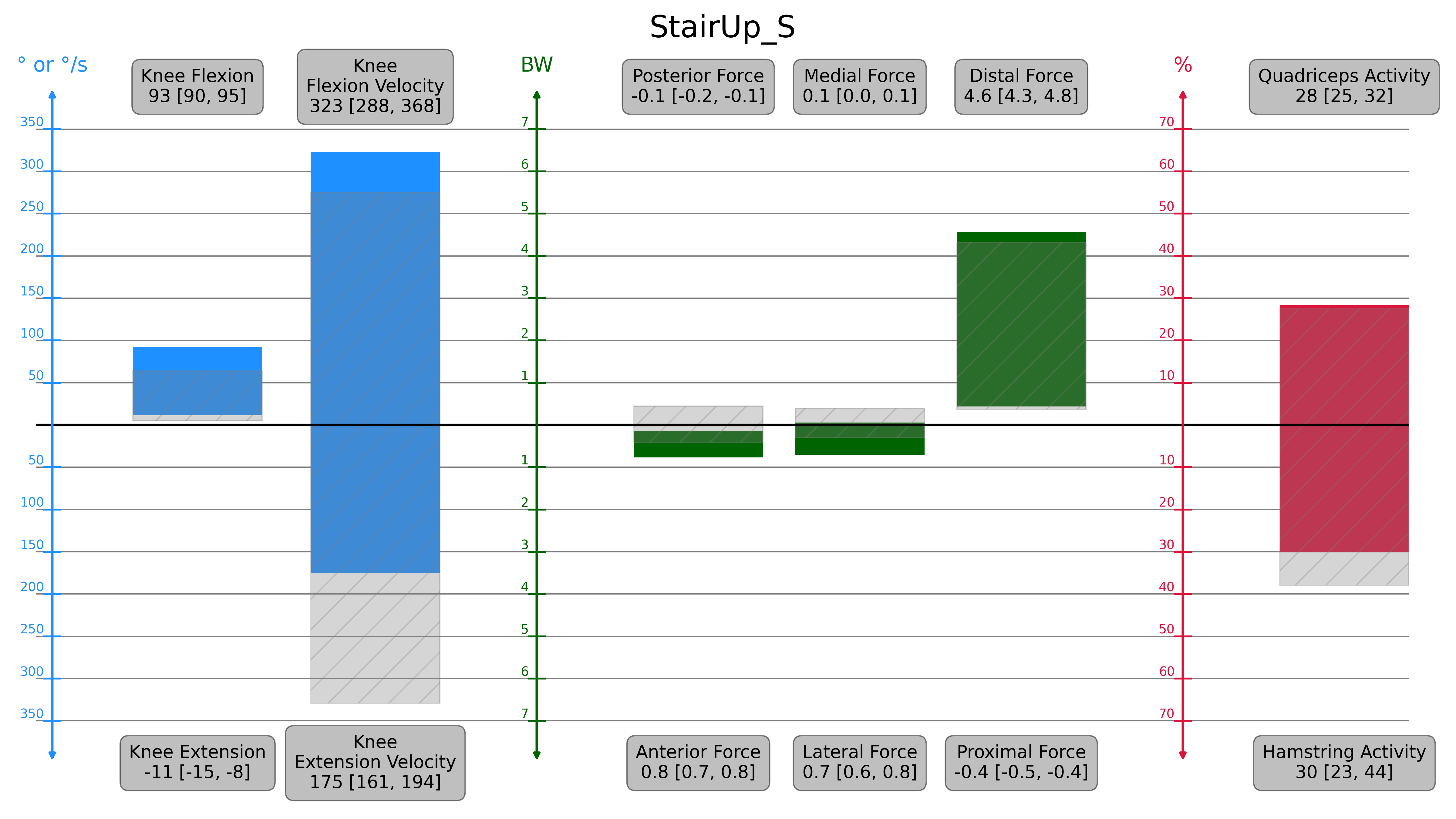

Supplement: sj-zip-1-thc-10.1177_09287329251413413 - Supplemental material for Comparing kinematic and kinetic demands on the knee joint during selected physiotherapy exercises and activities of daily living [file sj-zip-1-thc-10.1177_09287329251413413.zip › Task_StairUp_S.png]

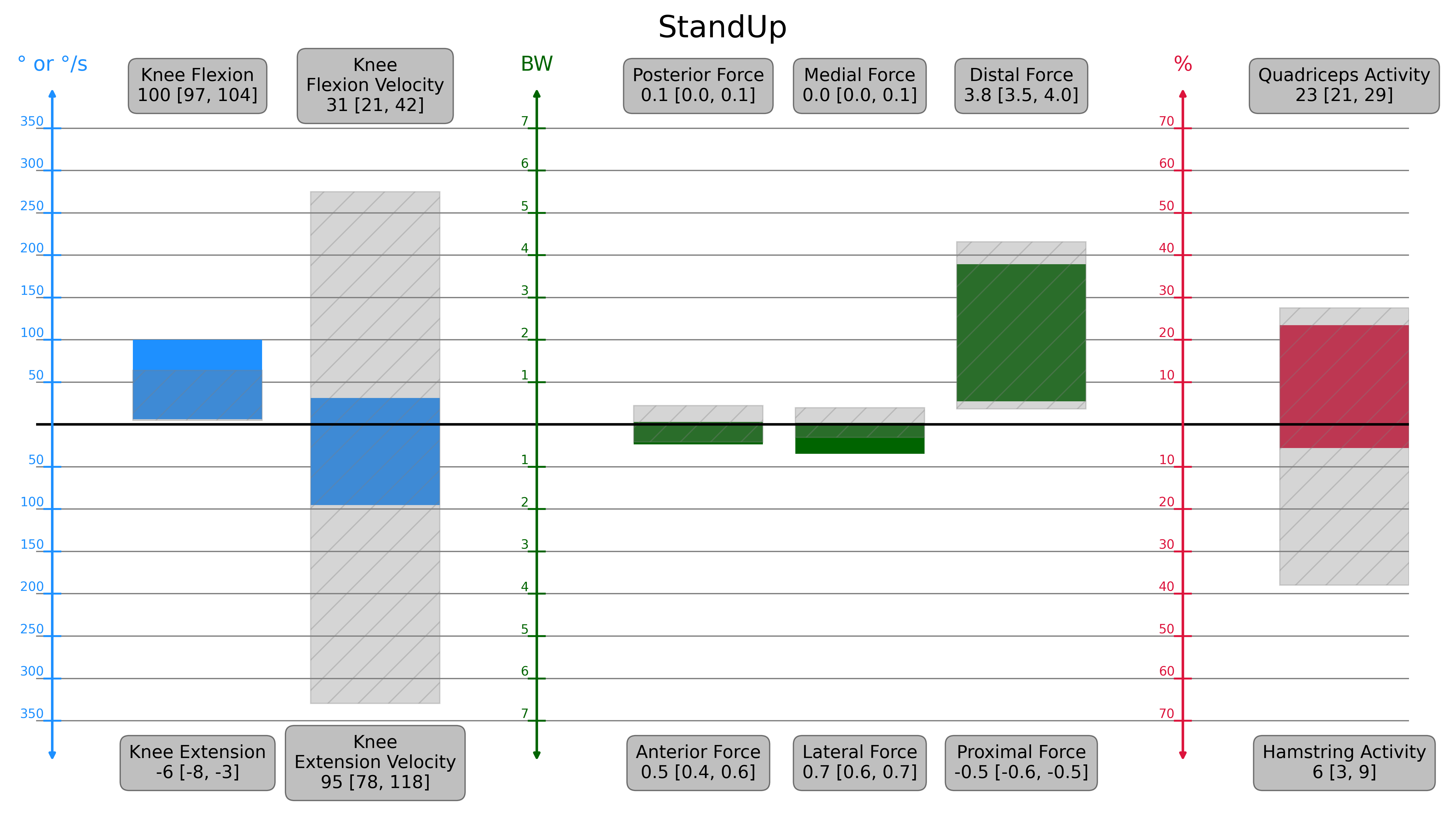

Supplement: sj-zip-1-thc-10.1177_09287329251413413 - Supplemental material for Comparing kinematic and kinetic demands on the knee joint during selected physiotherapy exercises and activities of daily living [file sj-zip-1-thc-10.1177_09287329251413413.zip › Task_StandUp.png]

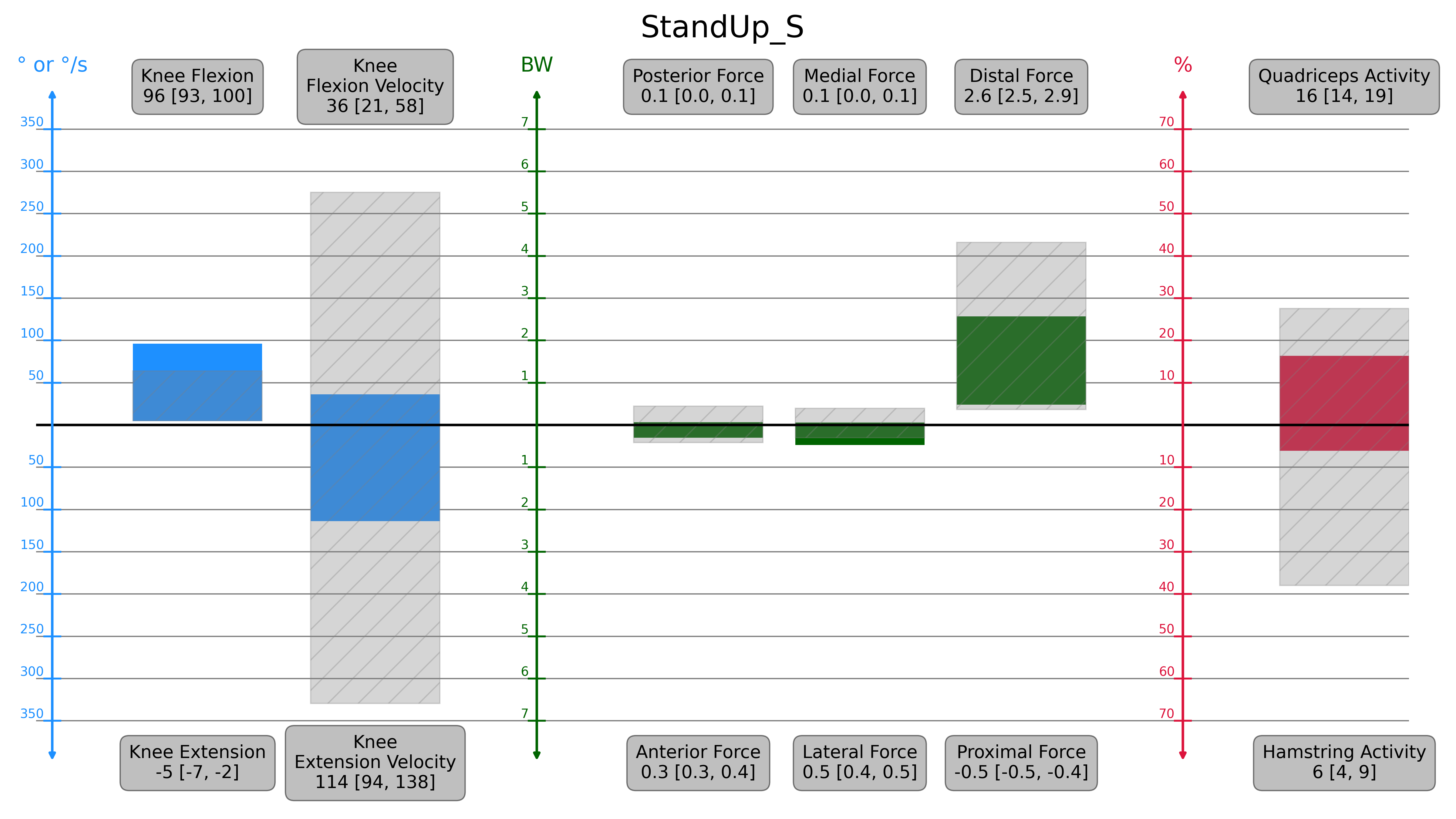

Supplement: sj-zip-1-thc-10.1177_09287329251413413 - Supplemental material for Comparing kinematic and kinetic demands on the knee joint during selected physiotherapy exercises and activities of daily living [file sj-zip-1-thc-10.1177_09287329251413413.zip › Task_StandUp_S.png]

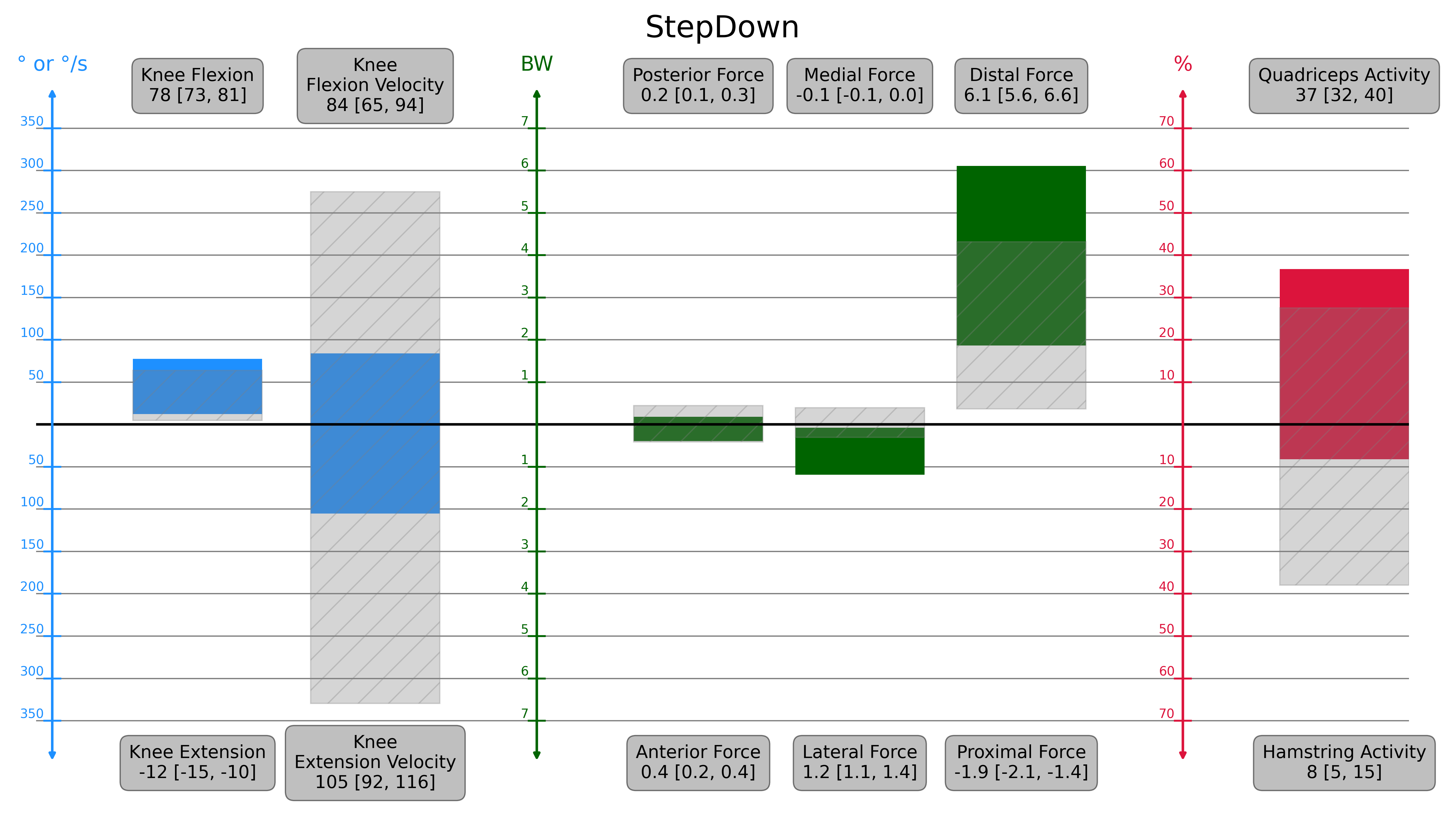

Supplement: sj-zip-1-thc-10.1177_09287329251413413 - Supplemental material for Comparing kinematic and kinetic demands on the knee joint during selected physiotherapy exercises and activities of daily living [file sj-zip-1-thc-10.1177_09287329251413413.zip › Task_StepDown.png]

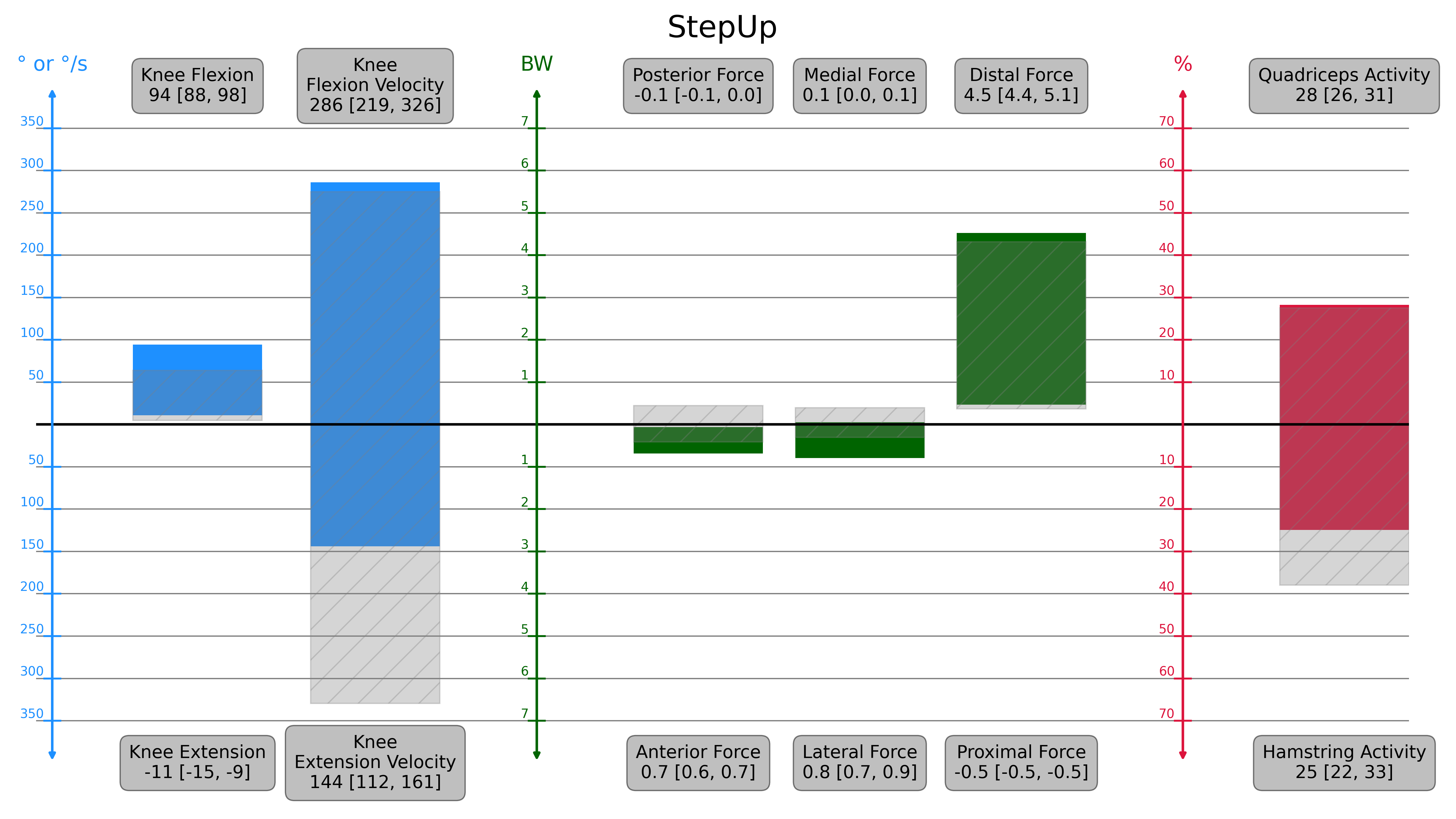

Supplement: sj-zip-1-thc-10.1177_09287329251413413 - Supplemental material for Comparing kinematic and kinetic demands on the knee joint during selected physiotherapy exercises and activities of daily living [file sj-zip-1-thc-10.1177_09287329251413413.zip › Task_StepUp.png]

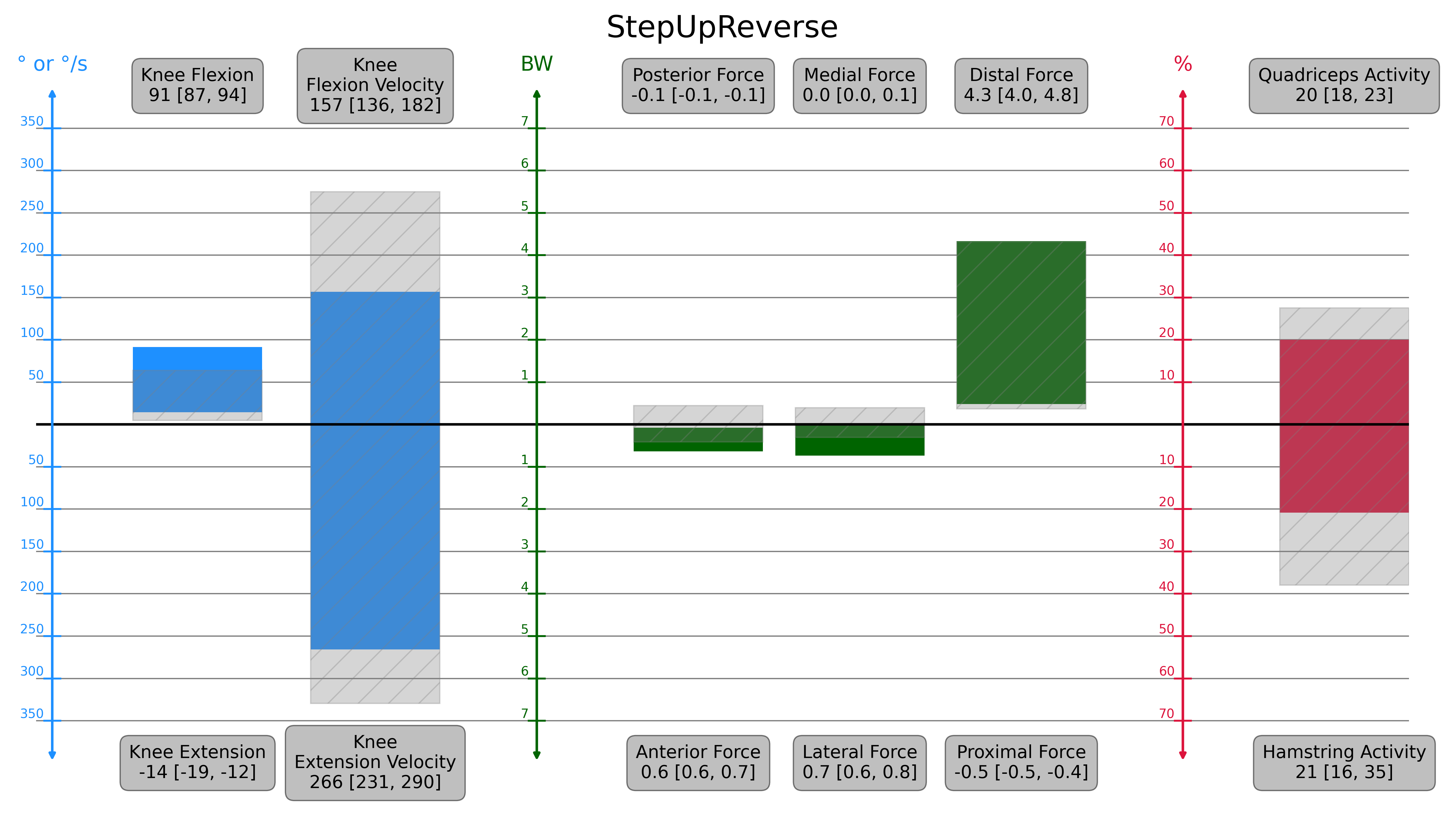

Supplement: sj-zip-1-thc-10.1177_09287329251413413 - Supplemental material for Comparing kinematic and kinetic demands on the knee joint during selected physiotherapy exercises and activities of daily living [file sj-zip-1-thc-10.1177_09287329251413413.zip › Task_StepUpReverse.png]

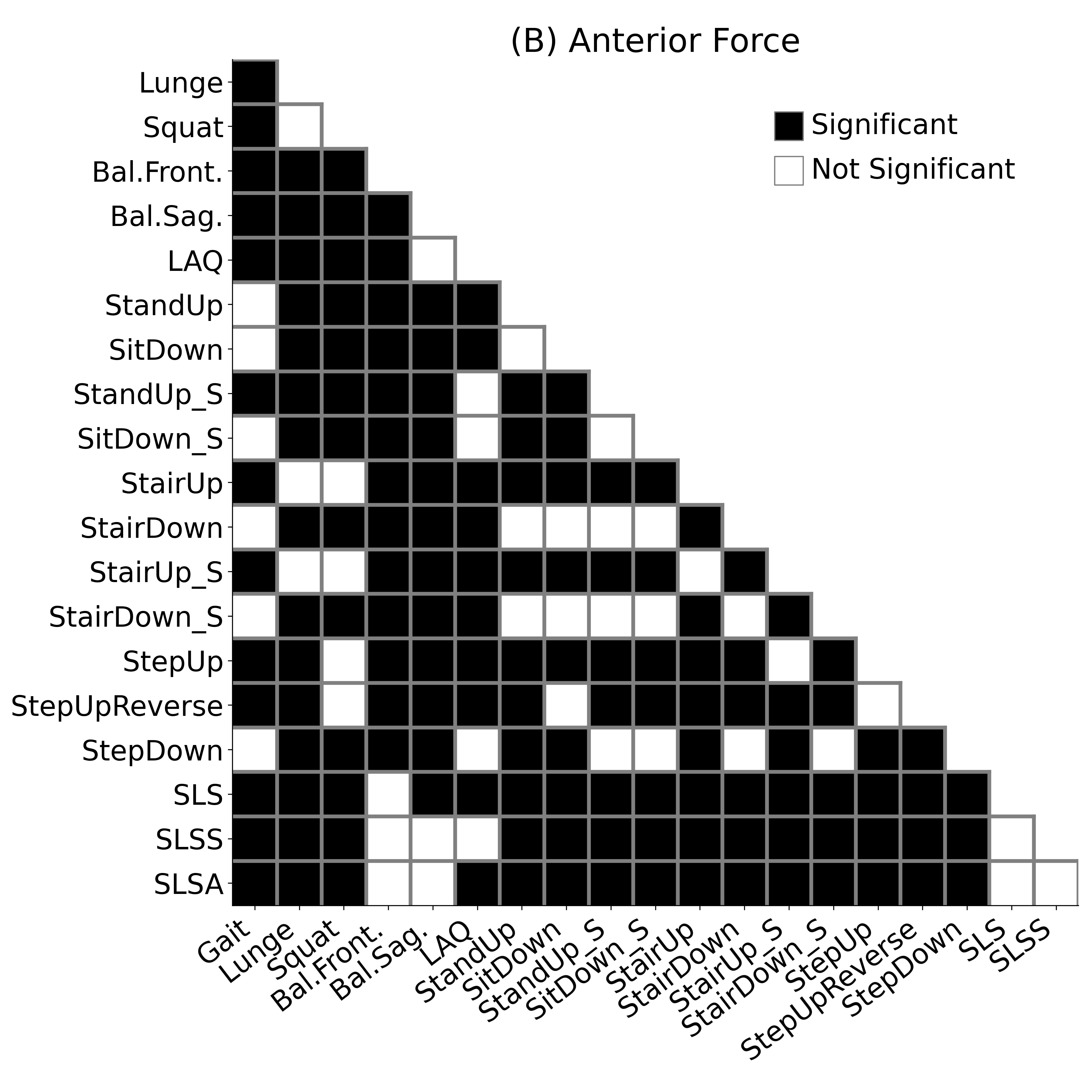

Supplement: sj-zip-1-thc-10.1177_09287329251413413 - Supplemental material for Comparing kinematic and kinetic demands on the knee joint during selected physiotherapy exercises and activities of daily living [file sj-zip-1-thc-10.1177_09287329251413413.zip › Comparison Anterior Force.png]

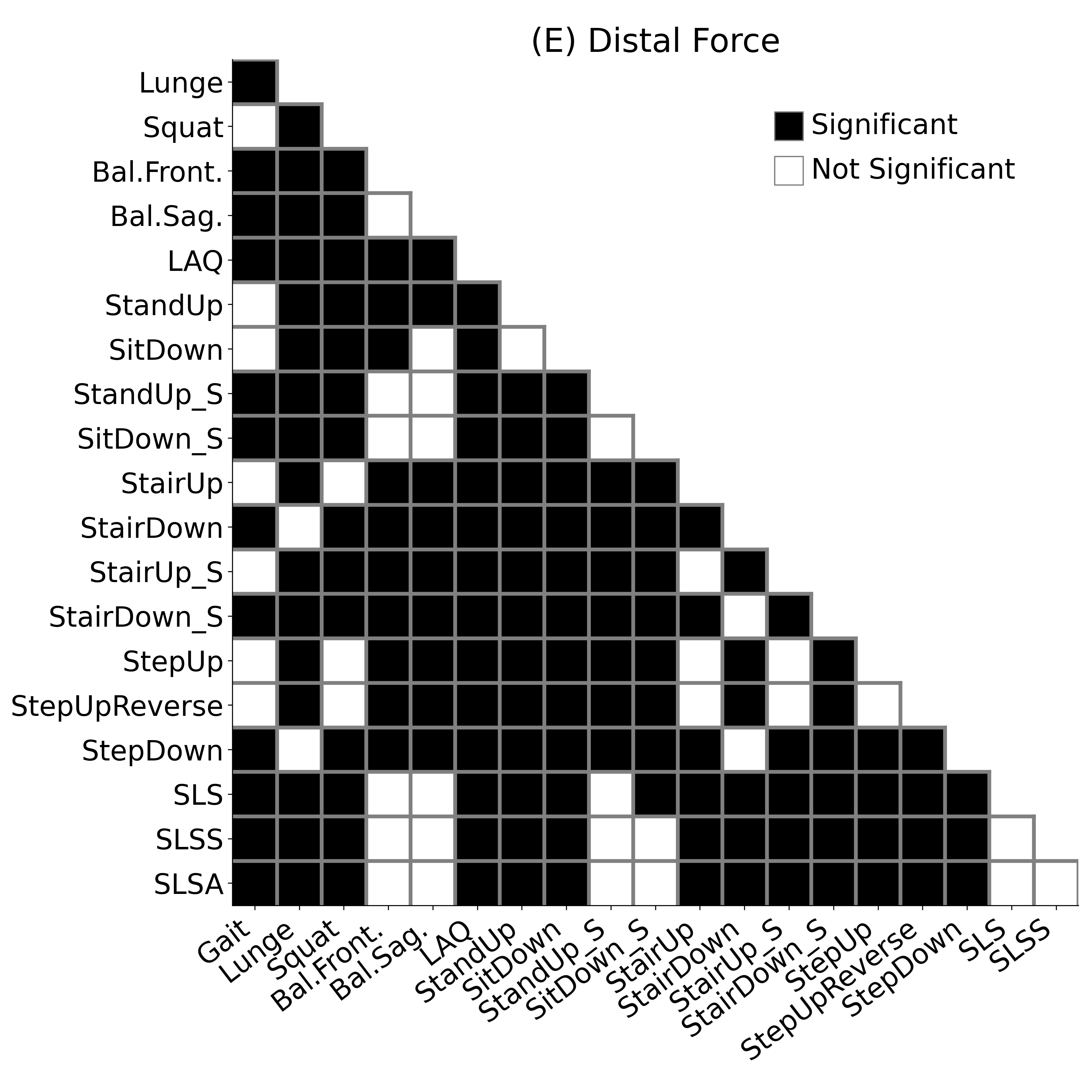

Supplement: sj-zip-1-thc-10.1177_09287329251413413 - Supplemental material for Comparing kinematic and kinetic demands on the knee joint during selected physiotherapy exercises and activities of daily living [file sj-zip-1-thc-10.1177_09287329251413413.zip › Comparison Distal Force.png]

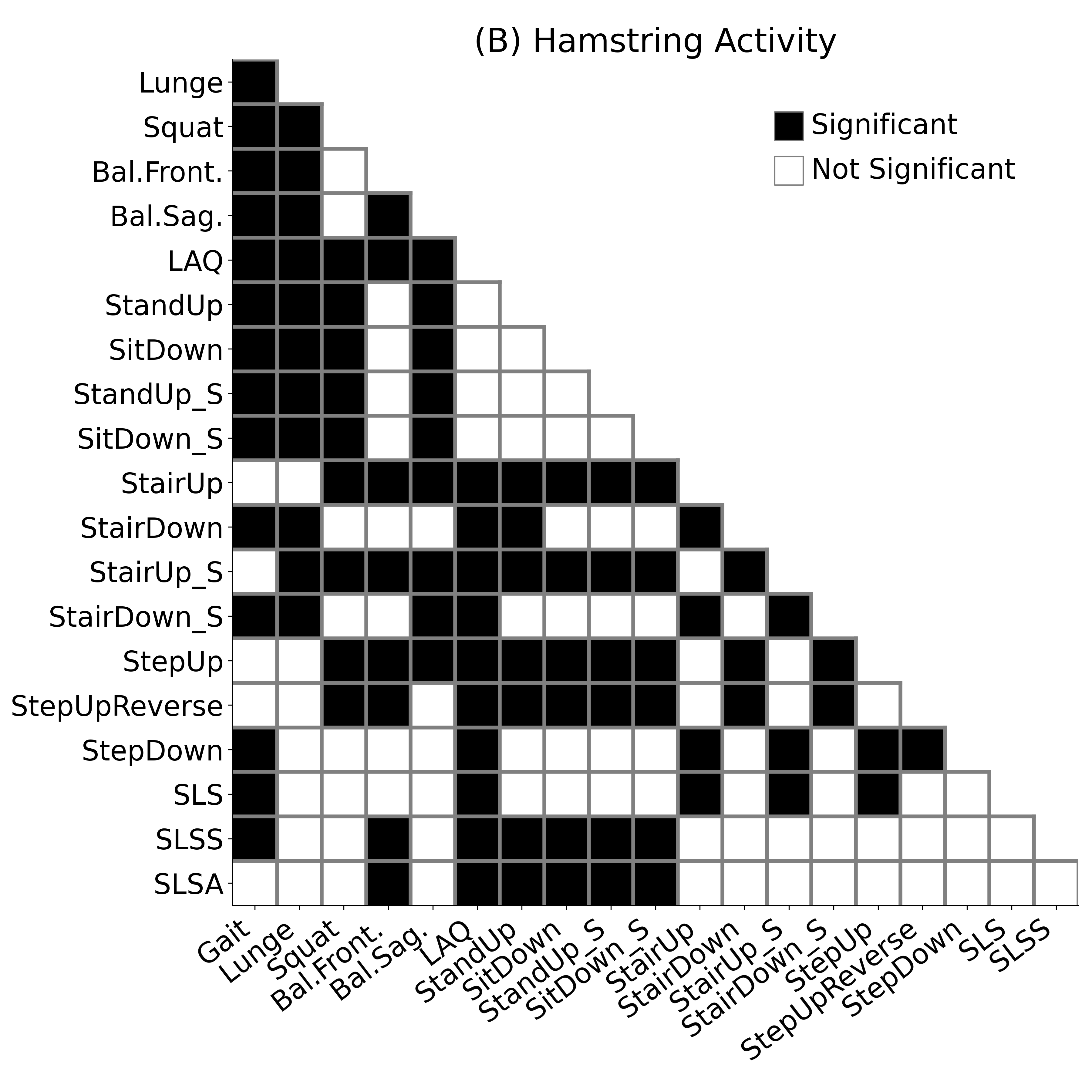

Supplement: sj-zip-1-thc-10.1177_09287329251413413 - Supplemental material for Comparing kinematic and kinetic demands on the knee joint during selected physiotherapy exercises and activities of daily living [file sj-zip-1-thc-10.1177_09287329251413413.zip › Comparison Hamstring Activity.png]

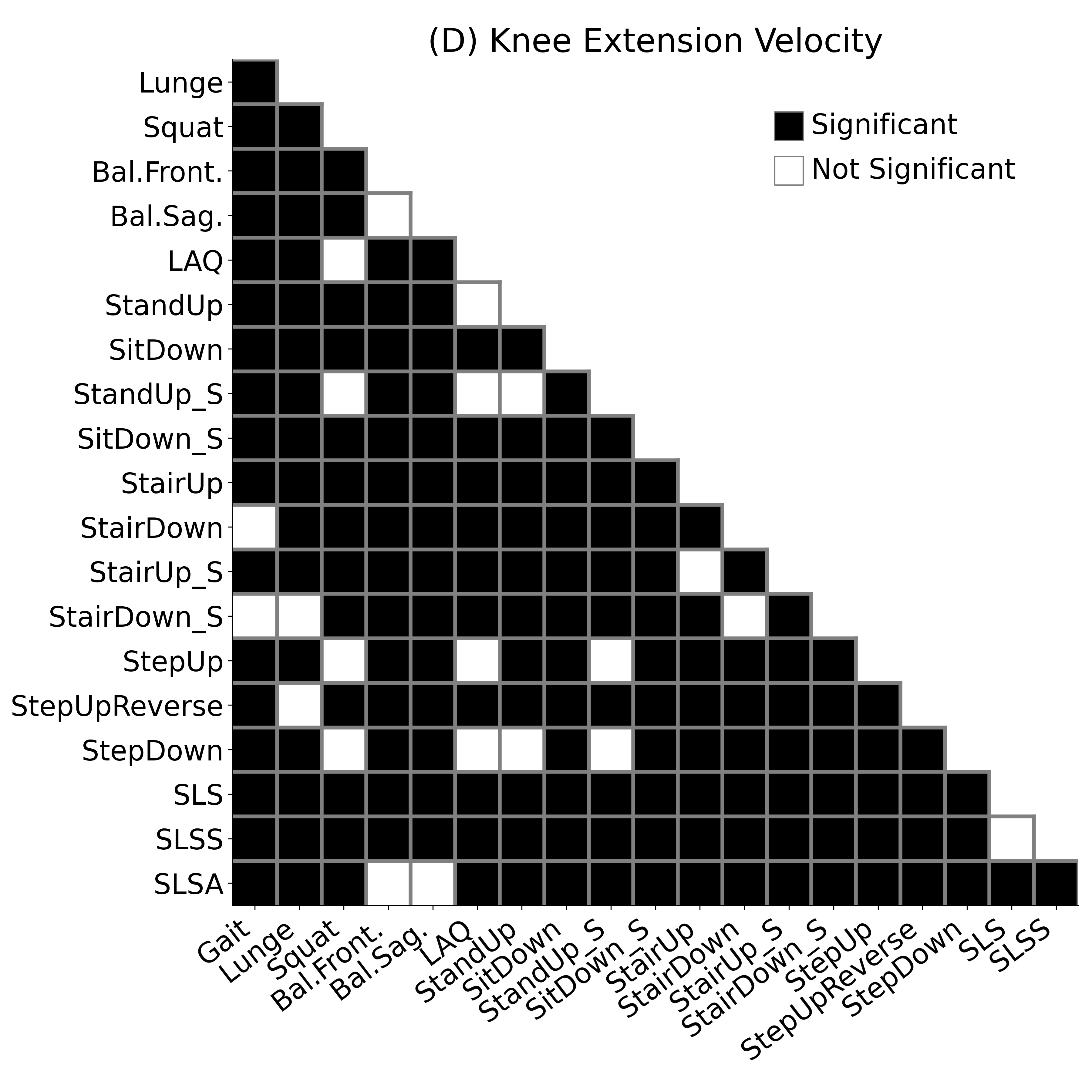

Supplement: sj-zip-1-thc-10.1177_09287329251413413 - Supplemental material for Comparing kinematic and kinetic demands on the knee joint during selected physiotherapy exercises and activities of daily living [file sj-zip-1-thc-10.1177_09287329251413413.zip › Comparison Knee Extension Velocity.png]

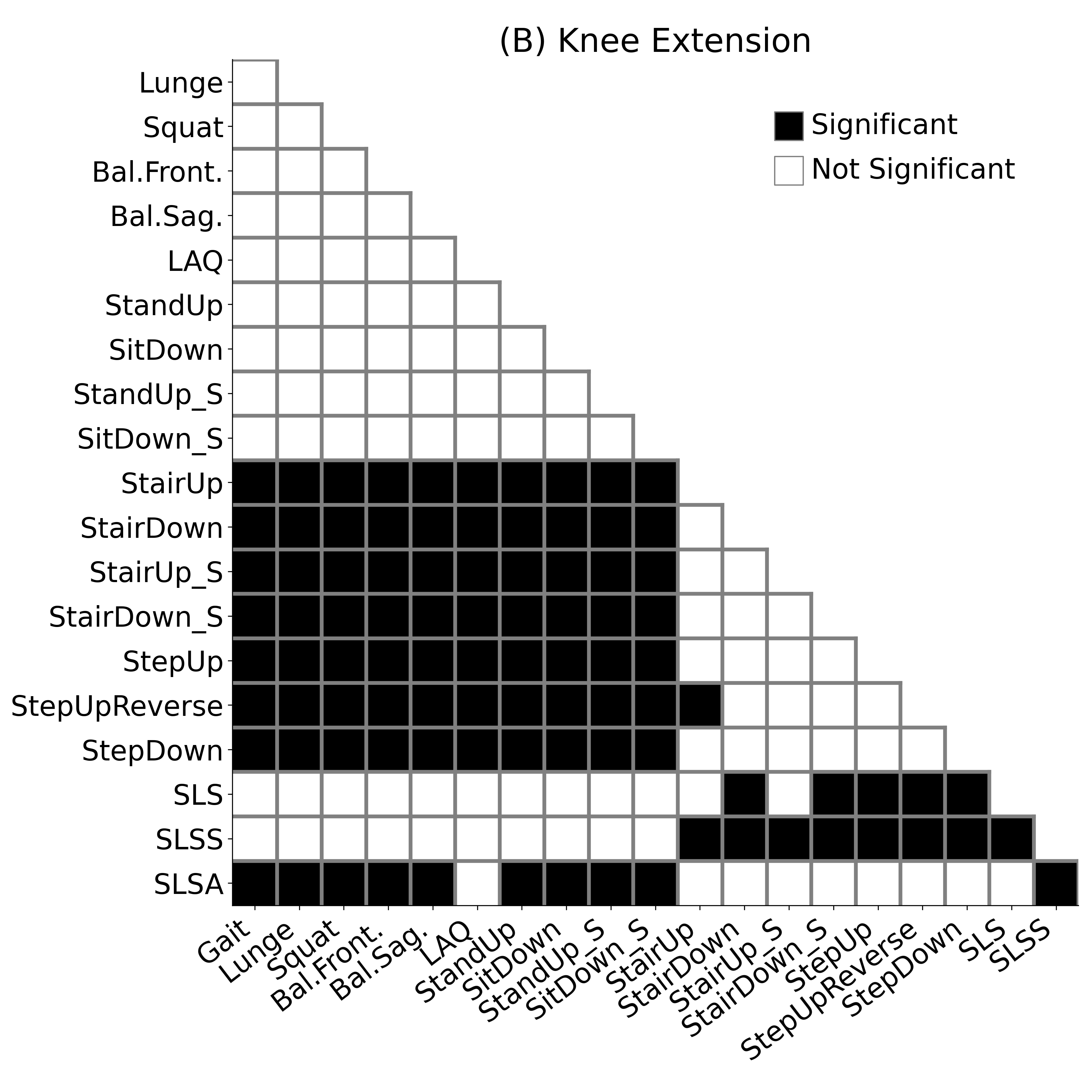

Supplement: sj-zip-1-thc-10.1177_09287329251413413 - Supplemental material for Comparing kinematic and kinetic demands on the knee joint during selected physiotherapy exercises and activities of daily living [file sj-zip-1-thc-10.1177_09287329251413413.zip › Comparison Knee Extension.png]

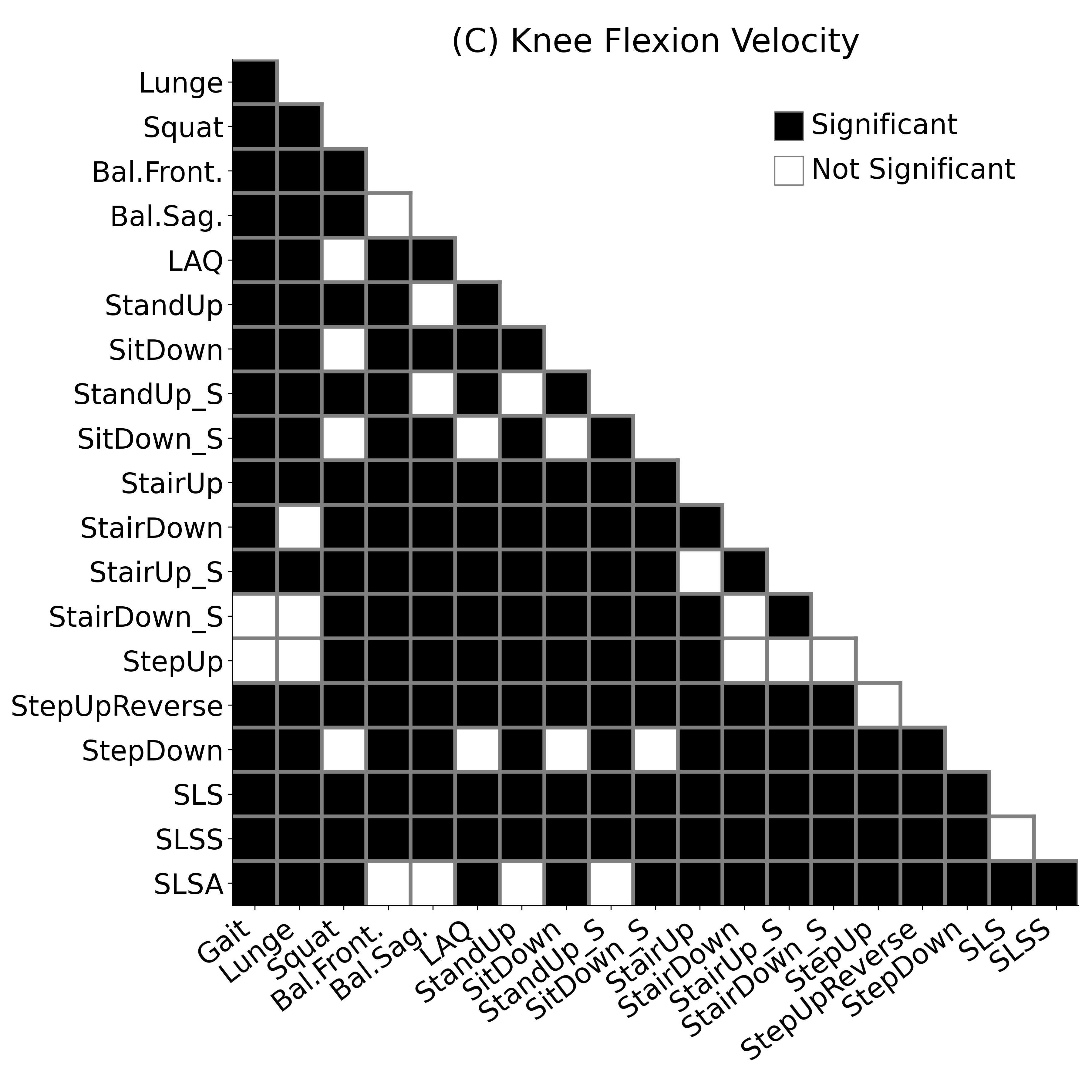

Supplement: sj-zip-1-thc-10.1177_09287329251413413 - Supplemental material for Comparing kinematic and kinetic demands on the knee joint during selected physiotherapy exercises and activities of daily living [file sj-zip-1-thc-10.1177_09287329251413413.zip › Comparison Knee Flexion Velocity.png]

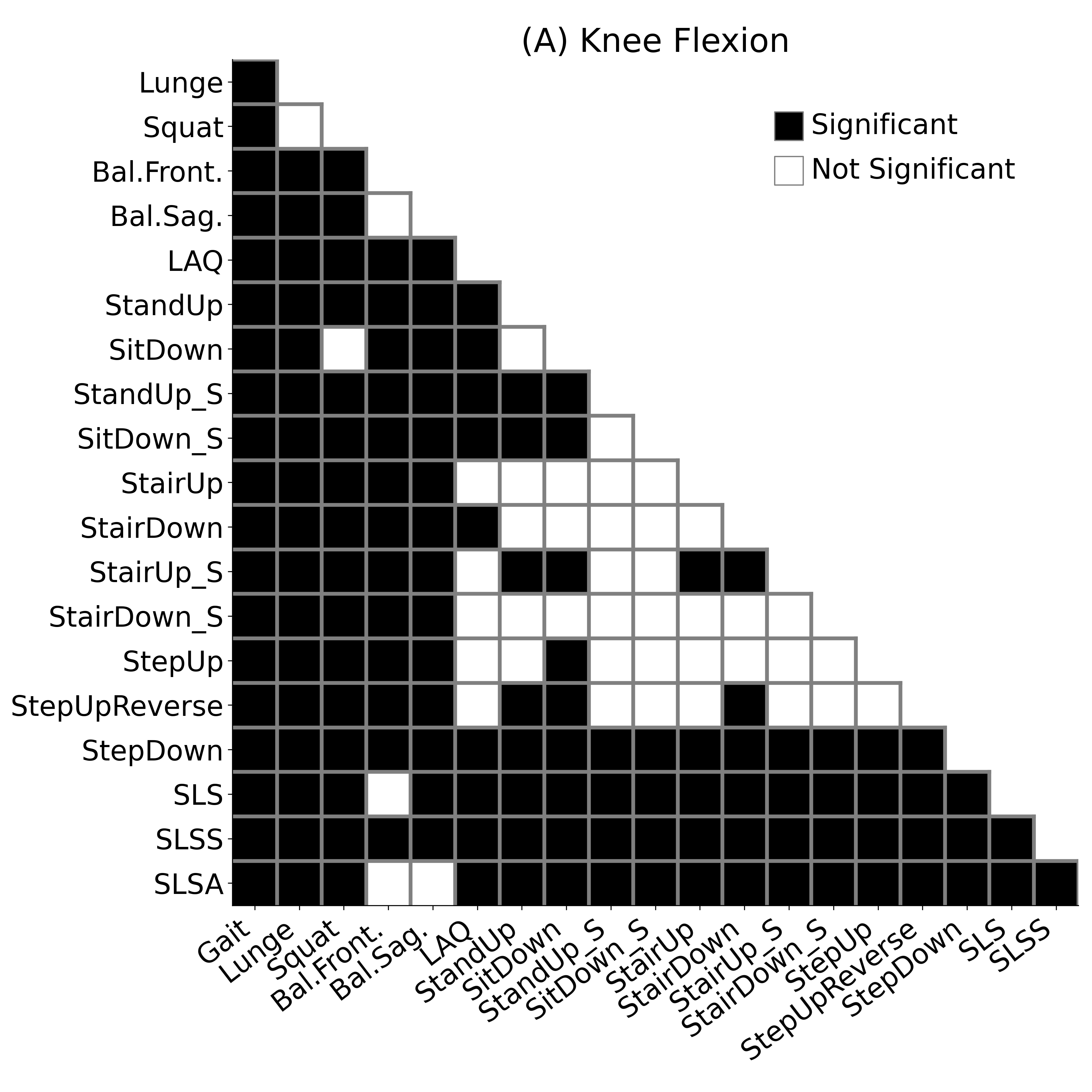

Supplement: sj-zip-1-thc-10.1177_09287329251413413 - Supplemental material for Comparing kinematic and kinetic demands on the knee joint during selected physiotherapy exercises and activities of daily living [file sj-zip-1-thc-10.1177_09287329251413413.zip › Comparison Knee Flexion.png]

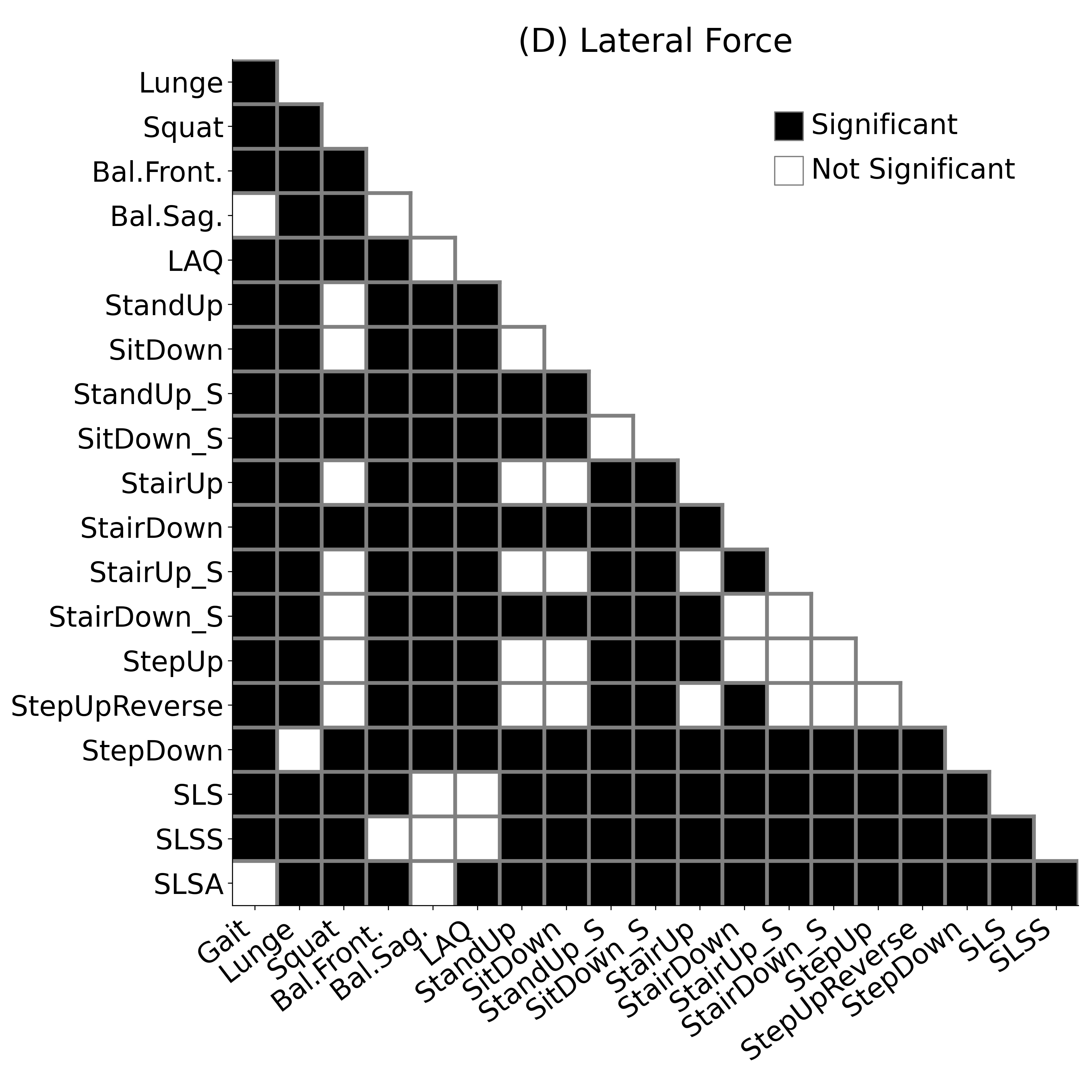

Supplement: sj-zip-1-thc-10.1177_09287329251413413 - Supplemental material for Comparing kinematic and kinetic demands on the knee joint during selected physiotherapy exercises and activities of daily living [file sj-zip-1-thc-10.1177_09287329251413413.zip › Comparison Lateral Force.png]

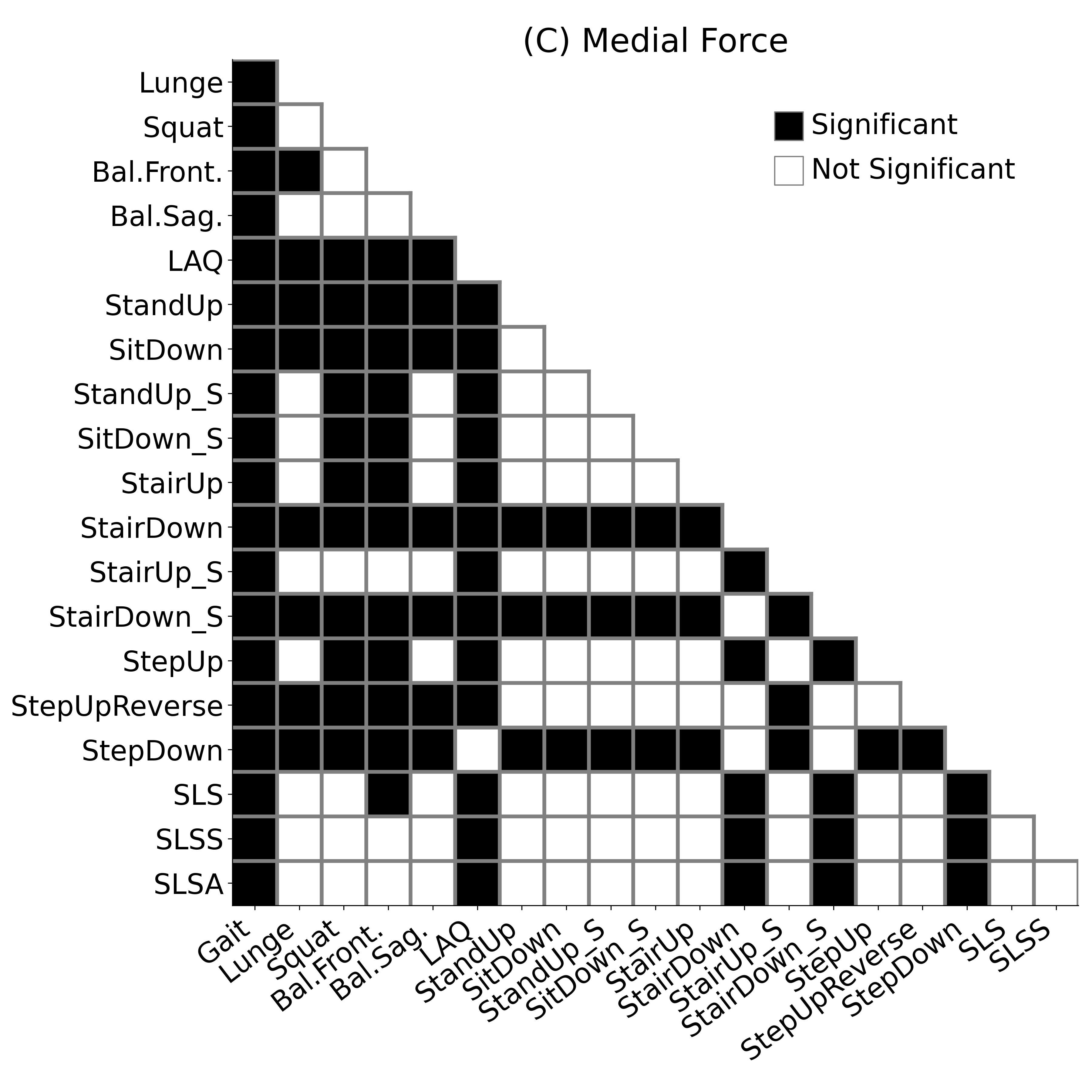

Supplement: sj-zip-1-thc-10.1177_09287329251413413 - Supplemental material for Comparing kinematic and kinetic demands on the knee joint during selected physiotherapy exercises and activities of daily living [file sj-zip-1-thc-10.1177_09287329251413413.zip › Comparison Medial Force.png]
